# Implementation of infection prevention and control in acute care hospitals in Mainland China – a systematic review

**DOI:** 10.1186/s13756-019-0481-y

**Published:** 2019-02-11

**Authors:** Jiancong Wang, Fangfei Liu, Jamie Bee Xian Tan, Stephan Harbarth, Didier Pittet, Walter Zingg

**Affiliations:** 1 Infection Control Program and WHO Collaborating Centre on Patient Safety, University of Geneva Hospitals and Faculty of Medicine, Rue Gabrielle Perret-Gentil 4, 1211 Geneva 14, Switzerland; 2 Institute of Global Health, Faculty of Medicine, University of Geneva, Geneva, Switzerland; 3 Department of Infection Control, Dong Guan Hospital of Traditional Chinese Medicine, Dong Guan City, Guang Dong Province China; 4 Department of Nosocomial Infection Management, The Second Affiliated Hospital, Xi’an Jiaotong University, Xi’an, Shaanxi Province China; 5 Department of Microbiology, Singapore General Hospital, Singapore, Singapore; 6 National Institute for Health Research Health Protection Research Unit in Healthcare Associated Infections and Antimicrobial Resistance, Imperial College of London, London, UK

**Keywords:** Healthcare-associated infection, Infection prevention and control, Hospital management, Systematic review, China, Adoption, Implementation

## Abstract

**Background:**

Healthcare-associated infections (HAIs) and antimicrobial resistance (AMR) affect patients in acute-care hospitals worldwide. No systematic review has been published on adoption and implementation of the infection prevention and control (IPC) key components. The objective of this systematic review was to assess adoption and implementation of the three areas issued by the “National Health Commission of the People’s Republic of China” in acute-care hospitals in Mainland China, and to compare the findings with the key and core components on effective IPC, issued by the European Centre for Disease Prevention and Control (ECDC) and the World Health Organization (WHO).

**Methods:**

We searched PubMed and the Chinese National Knowledge Infrastructure for reports on the areas “structure, organisation and management of IPC”, “education and training in IPC”, and “surveillance of outcome and process indicators in IPC” in acute-care facilities in Mainland China, published between January 2012 and October 2017. Results were stratified into primary care hospitals and secondary/tertiary care hospitals.

**Results:**

A total of 6580 publications were retrieved, of which 56 were eligible for final analysis. Most of them were survey reports (*n* = 27), followed by observational studies (*n* = 17), and interventional studies (*n* = 12), either on hand hygiene promotion and best practice interventions (*n* = 7), or by applying education and training programmes (*n* = 5). More elements on IPC were reported by secondary/tertiary care hospitals than by primary care hospitals. Gaps were identified in the lack of detailing on organisation and management of IPC, education and training activities, and targets of surveillance such as central line-associated bloodstream infections, ventilator associated pneumonia, catheter-associated urinary tract infections, and *Clostridium difficile* infections. Information was available on adoption and implementation of 7 out of the 10 ECDC key components, and 7 out of the 8 WHO core components.

**Conclusion:**

To variable degrees, there is evidence on implementation of all NHCPRC areas and of most of the ECDC key components and the WHO core components in acute care hospitals in Mainland China. The results are encouraging, but gaps in effective IPC were identified that may be used to guide future national policy-making in Mainland China.

**Electronic supplementary material:**

The online version of this article (10.1186/s13756-019-0481-y) contains supplementary material, which is available to authorized users.

## Introduction

The prevention of healthcare-associated infections (HAIs) is a first priority for patient safety in acute-care hospitals worldwide [1–5]. Adherence to the key and core components of infection prevention and control (IPC) issued by the European Centre for Disease Prevention and Control (ECDC)-funded “Systematic Review and Evidence-based Guidance on Organisation of Hospital Infection Control” (SIGHT) group and the World Health Organization (WHO), respectively, contributes to prevent HAI and the spread of antimicrobial resistance [6, 7]. The United Nations Sustainable Development Goals highlighted the importance of IPC as a contributor to safe and effective high-quality health service delivery [7]. Furthermore, WHO intends to support countries in the development of their own national IPC programmes [7].

The Asia-Pacific region has been described as a geographic source for emerging infectious diseases, including multidrug-resistant organisms and pathogens with pandemic potential [8]. The People’s Republic of China is the largest economic body in the region and faces similar global health challenges towards HAI and emerging antimicrobial resistance, as other countries in the region [2, 3, 8]. Little is known on how hospitals prevent HAIs and control the spread of multidrug-resistant microorganisms in Mainland China; in particular, there is lack of information on the availability and the implementation of the ECDC key components for effective IPC.

In 2006, the National Health Commission of the People’s Republic of China (NHCPRC) published the “Nosocomial Infection Management Methods” (Decree No. 48), which are guidelines defining elements on the organisation of IPC at hospital level [9]. In 2018, hospital accreditation was linked to the NHCPRC elements by the “Accreditation regulation of control and prevention of healthcare-associated infection in hospitals” (WS/T 592–2018) [10]. The NHCPRC decree embraces three broad areas of IPC: 1) structure, organization and management of IPC; 2) education and training in IPC; and 3) outcome and process indicator surveillance in IPC.

The aim of this systematic review was to assess adoption and implementation of elements of the three NHCPRC areas by acute care hospitals in Mainland China, and to compare the findings with the ECDC key components and the WHO core components in IPC.

## Methods

### Search strategy

This systematic review followed the “Preferred Reporting Items for Systematic Review and Meta Analysis” (PRISMA) guidelines [11]. We searched PubMed, the “Chinese National Knowledge Infrastructure” database, and the Cochrane library for any relevant document. In addition, we looked for guidelines on the official websites of the NHCPRC and the regional Ministries of Health in Mainland China.

Primary outcomes were: reporting on adopting, implementing (having) or analysing elements of the three NHCPRC areas. Secondary outcomes were: reporting on change of indicators (e.g. HAI or hand hygiene) by applying IPC practices. The search terms addressed the three IPC areas specified by the NHCPRC for acute care hospitals: 1) structure, organization and management of IPC; 2) education and training of IPC; and 3) surveillance of process and outcome indicators relevant to IPC. Search terms and key words for PubMed and the “Chinese National Knowledge Infrastructure” are summarized in Additional file 1: Tables S1A and S1B.

### Inclusion/exclusion criteria

Any article was eligible for inclusion when all of the following criteria were met: 1) use of a quantitative, qualitative or combined (mixed-methods) method; 2) reporting on one of the primary and/or secondary outcomes; 3) publication between January 2012 and October 2017; and 4) publication either in English or Chinese. Articles were excluded if they met one of the following criteria: 1) conference papers, editorials, or letters; 2) duplicated results; 3) risk factor analysis without information on the use of any IPC practice; 4) non-acute healthcare setting; or 5) outbreak investigations.

### Data extraction

Title, abstract and full text review were performed by two individual researchers (JW, FL). Disagreements were resolved by consensus, and, when necessary, discussed with a third researcher (WZ). Data extraction was stratified by two hospital categories (primary care and secondary/tertiary care hospitals). Definitions on hospital categories are provided in Additional file 1: Table S2. Articles were further categorised as survey reports, observational studies or interventional studies. The following data were extracted from survey reports: title, authors, publication year, province, total number of hospitals, and the number of hospitals applying specific elements of the three NHCPRC areas. The following data were extracted from observational studies: title, author, publication year, province, study aim, setting, surveillance protocol, sample size, study duration, methodology, and outcome. The following data were extracted from interventional studies: title, authors, publication year, province, study aim, population, intervention, comparison, study design and outcome. Data extraction for interventional studies followed the “PICO” (population – intervention – comparison – outcome) concept [11]. Data were verified by cross-checking (JW, FL and JBXT). Survey reports and observational studies were quality assessed by using the “Strengthening the Reporting of Observational Studies in Epidemiology” (STROBE) checklist (Additional file 1: Tables S3A and S3B) [12]. Interventional studies were quality assessed by using the “Integrated quality Criteria for the Review Of Multiple Study designs” (ICROMS) checklist (Additional file 1: Tables S3C and S3D) [13]. Findings were stratified by the three NHCPRC areas, and compared with the ECDC key components [6], and the WHO core components [7].

### Statistical analysis

Frequencies of elements mentioned in the survey reports were calculated on hospital level (with the corresponding 95% confidence interval), and stratified by hospital category. The difference of each identified element between hospital categories was tested by Pearson’s *Chi-* Square test. Statistical analysis was performed using STATA version 14.0 (Stata Corporation, College Station, Texas, USA). Results of observational and interventional studies were summarized descriptively.

## Results

**Fig. 1:**
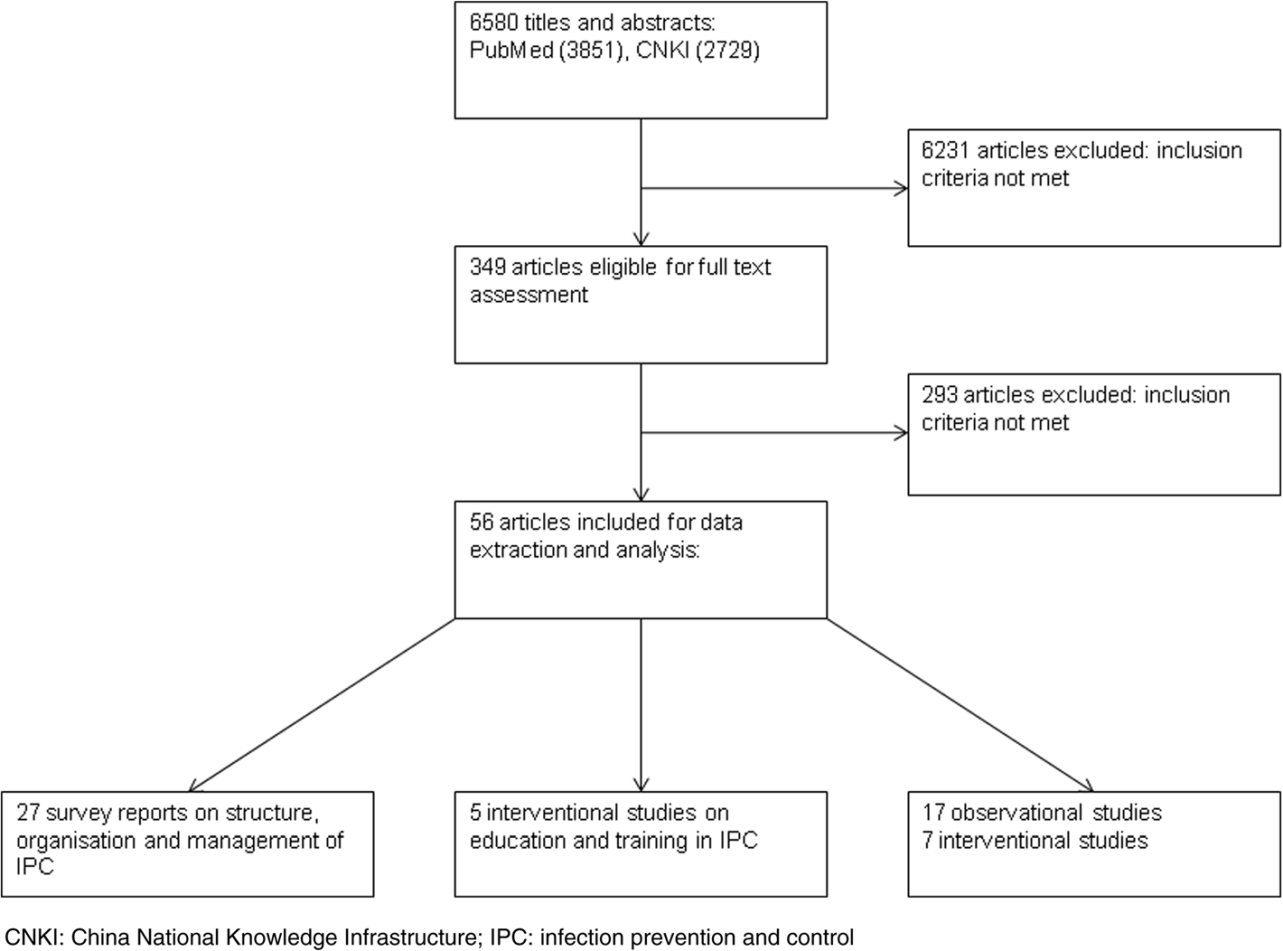
Systematic review profile – Systematic review on infection prevention and control in Mainland China, 2012–2017

From a total of 6580 titles and abstracts, 56 articles were eligible for data extraction and analysis (Fig. 1): 27 survey reports on structure, organisation and management of IPC (Table 1); 17 observational studies (8 single and 9 multicentre studies) measuring outcome and process indicators (Table 2); 5 interventional studies (5 single centre studies) applying education and training (Table 3); and 7 interventional studies (6 single- and 1 multicentre centre studies) testing the effectiveness of IPC strategies, mostly applying a multimodal strategy (*n* = 5) (Table 4).

**Table 1:** NHCPRC areas and elements of infection prevention and control identified by 27 survey reports – Systematic review on implementation of infection prevention and control in acute care hospitals in Mainland China, 2012–2017

NHCPRC areas	Elements	Primary care hospitals			Secondary/tertiary care hospitals			*P* value
		Reports (N)	Hospitals (N)	Yes^a^ N; % (95% CI)	Reports (N)	Hospitals (N)	Yes^a^ N; % (95% CI)	
Structure & organisation	Guideline provision	7	397	229; 57.7 (52.7–62.6)	6	492	422; 85.8 (82.4–88.7)	< 0.001
	Interdisciplinary IPC committee	6	360	256; 71.1 (66.1–75.7)	10	882	865; 98.1 (96.9–98.9)	< 0.001
	Formal IPC programme	5	302	187; 61.9 (56.2–67.4)	12	893	761; 85.2 (82.7–87.5)	< 0.001
	Feedback of IPC indicators	–	–	–	3	312	292; 93.6 (90.3–96.0)	–
	Allocated IPC funding/budget	–	–	–	3	282	86; 30.5 (25.2–36.2)	–
	IPC research	–	–	–	5	464	126; 27.2 (23.2–31.4)	–
Education & training	Postgraduate IPC training	8	379	203; 53.6 (48.4–58.7)	8	374	283; 75.7 (71.0–79.9)	< 0.001
Surveillance & Audit	Point prevalence survey of HAI	3	233	92; 39.5 (33.2–46.1)	3	201	135; 67.2 (60.2–73.6)	< 0.001
	Incidence surveillance of SSI	2	188	73; 38.8 (31.8–46.2)	5	406	292; 71.9 (67.3–76.2)	< 0.001
	Incidence surveillance in ICU	2	188	50; 26.6 (20.4–33.5)	5	406	157; 38.7 (33.9–43.6)	0.004
	Incidence surveillance in NICU	–	–	–	4	373	100; 26.8 (22.4–31.6)	–
	Surveillance of AMR	4	277	83; 30.0 (24.6–35.7)	7	459	295; 64.3 (59.7–68.7)	< 0.001
	Surveillance of antimicrobial use	4	231	129; 55.8 (49.2–62.4)	3	182	114; 62.6 (55.2–69.7)	0.164
	Standard and isolation precaution measures	5	201	81; 40.3 (33.5–47.4)	2	39	12; 30.8 (17.0–47.6)	0.264
	Waste management	9	423	266; 62.9 (58.1–67.5)	3	59	34; 57.6 (44.1–70.4)	0.435
	Sterilization and decontamination	7	372	217; 58.3 (53.1–63.4)	2	38	21; 55.3 (38.3–71.4)	0.715
	Environmental culturing	6	357	204; 57.1 (51.8–62.3)	3	201	186; 92.5 (88.0–95.8)	< 0.001
	Total	10^b^	466		19^b^	1168		

^a^Number of hospitals reporting on having established the element

^b^Two studies reported on both primary- and secondary/tertiary care hospitals

95% CI: 95% confidence interval; *AMR* antimicrobial resistance, *IPC* infection prevention and control, *ICU* intensive care unit, *NHCPRC* National Health Commission of the People’s Republic of China, *NICU* neonatal intensive care unit, *SSI* surgical site infection

**Table 2:** Observational studies in infection prevention and control – Systematic review on implementation of infection prevention and control in acute care hospitals in Mainland China, 2012–2017

Author, year, province	Study aim	Setting	Surveillance protocol	Sample size and study duration	Methodology	Outcome	Quality
Liu S, 2017, Jiangsu [60]	To investigate the association between ABHR use and HAI	Single centre	Research protocol	78,344 patients (January to December 2015)	Association between ABHR utilization and HAI incidence analysed by regression models	ABHR use was found to be negatively correlated with SSI incidence (hand sanitizer, r = − 0.85; soap, r = − 0.88; paper towels, r = − 0.83). Significant negative correlation between ABHR use and HAI in non-ICU patients (r = − 0.52 to – 0.65, *p* = 0.0032–0.029)	Moderate
Kang J, 2017, Multi-Region [42]	To determine the incidence of PICC-related complications in cancer patients	Multi-centre	Standard surveillance	477 cancer patients with 50,841 catheter-days (February 2013 to April 2014)	Prospective incidence surveillance	The incidence of CLABSI was 0.12 per 1000 catheter days	Moderate
Zhou H, 2017, Jiangsu [41]	To determine the HAI incidence in the ICUs of STCHs in one province	Multi-centre	Surveillance in a network	396,283 patients (July 2010 to June 2015)	Prospective incidence surveillance	The overall HAI incidence was 7.23%; VAP ID: 13.77 per 1000 ventilator days, CLABSI ID: 1.74 per 1000 central catheter days; CAUTI ID: 2.08 per 1000 urinary catheter days	High
Chen W, 2016, Jiangsu [39]	To determine (infection-associated) VAC incidence in adult ICU patients	Single centre	Standard surveillance	1014 patients (January to March 2015)	Prospective incidence surveillance	Of 197 patients on mechanical ventilation for a total of 3152 ventilator-days, 46 VACs were identified including 22 classified as infection-related (iVAC; 14.59 and 6.98 per 1000 ventilation days, respectively)	High
Lv T, 2016, Shanghai [38]	To determine the incidence of device-associated HAI in the NICU	Multi-centre	Standard surveillance	The number of patients was not reported (July to December 2014)	Prospective incidence surveillance	VAP ID was 3.78 cases per 1000 ventilator days, CLABSI ID was 1.63 cases per 1000 central catheter days	Moderate
Li C, 2015, Zhejiang [61]	To investigate the impact of hour of surgery on SSI in patients undergoing colorectal cancer surgery	Single centre	Standard surveillance	756 patients (January to December in 2014)	Surgery start time: T1: 07:00 to 12:00; T2: 12:01 to 18:00; T3: 18:01 to 24:00	SSI incidence was 14.5, 15.3, and 17.5% in groups T1, T2, and T3. The surgery operation timing did not appear to have any effect on the occurrence of SSI	Moderate
Zhu S, 2015, Sichuan [37]	To determine the incidence of VAEs	Multi-centre	Standard surveillance	5256 patients (April to July 2013)	Prospective incidence surveillance	VAEs ID were 11.1 per 1000 ventilator days (94 cases); this included 31 patients with iVAC (3.7 per 1000 ventilator days) and 16 with possible VAP	High
Peng H, 2015, Anhui [40]	To determine HAI incidence in the ICU	Single centre	Standard surveillance	4013 patients (January 2010 to December 2014)	Prospective incidence surveillance	HAI incidence:10.64%; Device-associated HAI incidence: 9.567 per 1000 bed days; VAP ID: 19.561 per 1000 mechanical ventilator days; CLABSI ID: 2.716 per 1000 central line days; CAUTI ID: 1.508 per 1000 urinary-catheter days	High
Liu W, 2015, Inner Mongolia [36]	To determine HAI incidence in the ICU	Multi-centre	Standard surveillance	7255 patients (January to December 2013)	Prospective incidence surveillance	VAP ID: 10.02 per 1000 mechanical ventilator days; CLABSI ID: 1.56 per 1000 central catheter days; CAUTI ID: 2.26 per 1000 urinary catheter-days	Moderate
Huang H, 2014, Shanghai [46]	To determine CDI incidence, and assess associated risk factors	Single centre	Standard surveillance	240 patients with hospital-acquired diarrhoea (September 2008 to April 2009)	Prospective incidence surveillance	90 patients (37.5%) (128.5 per 100,000 patient-days) with CDI (12 due to recurrent disease)	Moderate
Zhou F, 2014, Shanghai [45]	To identify clinical characteristics of CDI in patients with antibiotic-associated diarrhoea	Single centre	Standard surveillance	20,437 patients (August 2012 to July 2013)	Prospective incidence surveillance	Antibiotic-associated diarrhoea developed in 1.0% (206 patients) of patients receiving at least one dose of antibiotics; *C. difficile* was isolated from 30.6% (63) of patients with antibiotic-associated diarrhoea	Moderate
Wang X, 2014, Si Chuan [44]	To investigate the incidence, clinical profiles and outcome of ICU-onset CDI	Single centre	Standard surveillance	1277 patients (May 2012 to January 2013)	Prospective incidence surveillance	124 patients with ICU-onset diarrhoea; 31 patients with CDI (252 cases per 100,000 ICU days)	High
Peng S, 2013, Liaoning [43]	To determine the incidence, risk factors and outcomes of CRBSI in the ICU	Single centre	Standard surveillance	174 patients (June 2007 to May 2008)	Prospective incidence surveillance	21 patients developed CRBSI (11.0 per 1000 central catheter days with a catheter utilization rate of 72.8%)	High
Hu B, 2013, Multi-region [35]	To determine device-associated HAIs, in ICUs	Multi-centre	Surveillance in a network	2631 patients (August 2008 to July 2010)	Prospective incidence surveillance	VAP ID: 10.46 per 1000 ventilator-days; CLABSI ID: 7.66 per 1000 central line-days; CAUTI ID: 1.29 per 1000 urinary catheter-days	High
Xu C, 2013, Hubei [34]	To determine the HAI incidence in the ICUs of Hubei Province	Multi-centre	Surveillance in a network	20,641 patients (January to December 2010)	Prospective incidence surveillance	CLABSI ID: 1.40 per 1000 central catheter days; VAP ID: 30.82 per 1000 ventilator days; CAUTI ID: 1.50 per 1000 urinary catheter days	Moderate
Liu Y, 2012, Multi-region [33]	To investigate aetiology and incidence of HAP	Multi-centre	Surveillance in a network	42,877 patients (August 2008 to December 2010)	Prospective incidence surveillance	610 HAP with an incidence of 1.4% (0.9% in the respiratory general ward, 15.3% in the respiratory ICU)	Moderate
Liu K, 2012, Beijing [32]	To determine device-associated HAIs in the ICUs of tertiary-care hospitals	Multi-centre	Standard surveillance	ICUs of 38 tertiary care hospitals in Beijing (no study duration reported)	Prospective incidence surveillance	CRBSI ID: 2.5 per 1000 central catheter days; CAUTI ID: 2.1 per 1000 urinary catheter days; VAP ID: 7.6 per 1000 ventilator days	Moderate

*ABHR* alcohol-based handrub, *CAUTI* catheter-associated urinary tract infection, *CDI Clostridium difficile* infection, *CLABSI* central line-associated bloodstream infection, *CRBSI* catheter-related bloodstream infection, *HAI* healthcare-associated infection, *HAP* hospital-acquired pneumonia, *HH* hand hygiene, *ICU* intensive care unit, *ID* incidence density, *NICU* neonatal intensive care unit, *PICC* Peripherally inserted central venous catheter, *SSI* surgical site infection, *VAC* ventilator-associated condition, *VAE* ventilator-associated event, *VAP* ventilator-associated pneumonia

Note: standard surveillance refers to the use of the standard Chinese surveillance protocol [62]

**Table 3:** Interventional studies applying education and training in infection prevention and control – Systematic review on implementation of infection prevention and control in acute care hospitals in Mainland China, 2012–2017

Author, year, province	Study aim	Population	Intervention	Comparison	Study design	Outcome	Quality
Chen S, 2017, Yunnan [24]	To assess the effectiveness of IPC training delivered at morning shift meetings	Single centre; 239 healthcare workers (nurses and doctors)	IPC lectures delivered at morning shift meetings	Same group of HCWs	NCITS; knowledge tests before, immediately after, and 3 months after IPC training	Knowledge significantly improved from 45.1 to 96.7%, and 83.9% (*P* < 0.001)	High
He M, 2017, Fujian [25]	To assess the effectiveness of IPC training delivered to new employees	Single centre; 343 new employees in pre-job training (nurses and doctors)	Lectures, problem-based learning, group discussions, demonstrations of various procedures	Same group of HCWs	NCBA; knowledge test before and after training	Knowledge on IPC significantly improved from 29.15–58.02% before training to 63.56–92.13% after training (*P* < 0.01)	High
Zhang Y, 2016, Guangdong [26]	To assess the effectiveness of an enhanced IPC training programme on new employees	Single centre; 716 HCWs in intervention group;	Lectures, video scenarios, simulation training, and group discussion	445 HCWs in control group	CBA; knowledge test and competency assessments before and after training using a structured questionnaire	Scores on both IPC knowledge and practice improved after training (*P* < 0.05). Scores during intervention period were higher compared to the pre-intervention period (*P* < 0.05)	High
Huang M, 2014, Hebei [27]	To assess the effect of IPC training among nursing students on HH compliance	Single centre; 520 HH opportunities of 42 nursing students in the intervention group	8 h IPC training (video scenarios, on-site training, knowledge test)	518 HH opportunities of 38 nursing students in the control group	CBA; HH compliance of nursing students receiving and not receiving additional 8 h of IPC training one week after starting internship	HH compliance was significantly higher in the intervention group (74.2% vs. 46.7%; *P* < 0.01)	High
Zhao L, 2014, Guizhou [28]	To assess the effectiveness of IPC training in reducing HAI incidence	Single centre; 641 trained healthcare workers; 81 patients with HAI	Lectures, problem-based learning, on-site training, knowledge test	Same group of HCWs; 10,734 patients without HAI	NCBA; knowledge test and competency assessment before and after an IPC training programme; incidence of HAI before and during intervention	Higher knowledge and competency test scores after training. Significant reduction of HAI incidence from 1.26% in 2009 to 0.43% in 2012 (*P* < 0.05)	High

*CBA* Controlled before-after study, *HAI* healthcare-associated infection, *HH* hand hygiene, *IPC* infection prevention and control, *NCBA* non-controlled before-after study, *NCITS* non-controlled interrupted time-series analysis

**Table 4:** Interventional studies in infection prevention and control – Systematic review on implementation of infection prevention and control in acute care hospitals in Mainland China, 2012–2017

	Author, year, province	Study aim	Population	Intervention	Comparison	Study design	Outcome	Quality
Multimodal strategies	Mu X, 2016, Guizhou [53]	To assess the effectiveness of an intervention program on HH	Single centre; 26,586 HH opportunities in the intervention period	The intervention included improving HH facilities, education on HH, and quarterly reports on HH compliance and ABHR consumption	1266 HH opportunities during baseline	NCBA; quasi-experimental. Surveillance of HH compliance, ABHR consumption, use of paper towels	HH compliance improved from 37.78% at baseline to 75.90% after intervention (*P* < 0.001); ABHR consumption increased from 7.40 ml per patient-day at baseline to 12.15 ml after intervention (*P* = 0.004); paper towels use increased from 4.07 sheets per patient-day at baseline to 7.48 sheets after intervention (*P* < 0.001)	High
	Su D, 2015, Multi-region [54]	To assess the impact of INICC HH intervention	Multi-centre (5 ICUs of 3 hospitals); 1368 HH observations in interventional period	Administrative support; availability of ABHR and soap at the point of care; education and training on HH indications, reminders at the workplace, and HH surveillance with performance feedback	711 HH observations during baseline	NCBA. HH compliance during baseline and intervention	HH compliance increased from 51.5% during baseline to 80.1% during intervention (*P* = 0.004)	High
	Zhou Q, 2015, Shanghai [55]	To assess the impact of a CLABSI prevention programme	Single centre; 51 newborns in intervention; 91 newborns in follow-up	HH training; dedicated PICC team, all-inclusive central line cart, pre-packaged kits; daily evaluation of central line necessity; simulation training	29 newborns in pre-intervention	NCBA. CLABSI incidence density in baseline and intervention period	CLABSI ID decreased from 16.7 per 1000 central line-days at baseline to 7.6 in intervention (*P* = 0.08), and to 5.2 in follow-up (*P* < 0.01)	High
	Zhou Q, 2013, Shanghai [56]	To assess the efficacy of a VAP prevention programme in a NICU	Single centre; 169 neonates in partial intervention; 216 neonates in full intervention	HH training; waste disposal; isolation precaution measures; laminar airflow; use of ventilators (disinfection); reduction of ventilator- and antimicrobial days	106 neonates in pre-intervention	NCBA. VAP-incidence density surveillance	VAP ID decreased from 48.8 per 1000 ventilator-days in baseline to 25.7 in partial intervention, and to 18.5 in full intervention (*P* < 0.001)	High
	Tao L, 2012, Shanghai [57]	To assess the impact of a VAP prevention programme	Single centre; 3 ICUs (surgical, cardiothoracic, medical); 4112 patients in 2006; 4405 in 2007; 3992 in 2008; 3330 in 2009	Oral care with chlorhexidine twice daily, HH promotion, and semi-recumbent position	3250 patients during baseline (2005)	NCBA. Process and outcome surveillance (VAP incidence density) with feedback	VAP ID decreased from 24.1 per 1000 ventilator-days in 2005 to 16.6 in 2006, 9.5 in 2007, 7.5 in 2008, and 5.7 in 2009 (*P* = 0.0001)	High
Other IPC intervention	Li Q, 2017, Zhejiang [63]	To assess the impact of relocating a NICU and improving environmental cleaning on MRSA	Single centre; 800 environmental surface samples during intervention	Reprocessing microfiber cloths; disinfection of cots, incubators, screens, syringe pumps, carts, and isolation rooms	100 environmental surface samples during baseline	NCBA. MRSA in environmental surface samples	Significant decrease of MRSA-positive surfaces from 44.0% at baseline to 2.5% at intervention (*P* < 0.001)	High
	Lin Y, 2015, Fujian [64]	To evaluate the effect of chlorhexidine mouthwash before major heart surgery on VAP	Single centre; 47 patients	Gargling 3 × 30 s 30 min after each meal and 5 min after tooth brushing either with 0.2% chlorhexidine or normal saline on the day before major heart surgery	47 patients	RCT. Blind and random assignment of cardiac surgery patients to the 0.2% chlorhexidine or normal saline group	Significantly less VAP in the intervention group (8.5% vs. 23.4%; *P* = 0.049)	High

*ABHR* Alcohol-based handrub, *CLABSI* Central line-associated bloodstream infection, *HH* Hand hygiene, *ICU* Intensive care unit, *ID* incidence density, *INICC* International Nosocomial Infection Control Consortium, *IPC* Infection prevention and control, *MRSA* Methicillin-resistant *Staphylococcus aureus*, *NCBA* Non-controlled before-after study, *NICU* neonatal intensive care unit, *PICC* Peripherally inserted central venous catheter, *RCT* Randomised controlled trial, *VAP* Ventilator associated pneumonia

### NHCPRC area “structure, organisation and management of IPC”

The search terms addressing the NHCPRC area on “structure, organisation and management of IPC” identified 27 survey reports summarizing the results of 1634 hospitals: 8 (29.6%) reports on 440 primary care hospitals, 17 (63.0%) reports on 1127 secondary/tertiary care hospitals, and 2 (7.4%) reports on 26 primary- and 41 secondary/tertiary care hospitals combined (Table 1). The results of this area were divided into six elements (Table 1). Quality was moderate and low in eight and two of the 10 survey reports from primary care hospitals, respectively (Additional file 1: Table S4A). Quality was high, moderate and low in 1, 14, and 4 of the 19 survey reports from secondary/tertiary care hospitals, respectively (Additional file 1: Table S4B). Table 1, Additional file 1: Table S4A and Table S4B summarize the details on the reported elements, stratified by hospital types.

#### Structure, organisation and management, guideline provision

Most primary care hospitals had an IPC committee (71.1%), a formal IPC programme (61.9%), and provided IPC guidelines (57.7%). Most secondary/tertiary care hospitals had an IPC committee (98.1%), performed feedback on IPC indicators (93.6%), and provided IPC guidelines (85.8%). No information on feedback, allocated IPC funding/budget, and IPC research was identified for primary care hospitals. The frequencies of the elements were significantly different between hospital types, in favour for secondary/tertiary care hospitals. Only secondary/tertiary care hospitals reported numbers on IPC staff. The pooled ratios of IPC professionals, IPC doctors and IPC nurses were 0.51 (0.48–0.53), 0.13 (0.11–0.14), and 0.31 (0.28–0.33) per 100 beds, respectively.

#### Education and training in IPC

Significantly more secondary/tertiary care hospitals offered regular, postgraduate IPC training compared to primary care hospitals (Table 1). However, the survey reports did not describe details on target population, training content, or frequency of training activities.

#### Indicator and outcome surveillance in IPC

The results of this area were stratified by “surveillance” and “auditing” and divided into 10 elements (Table 1). Surveillance of antimicrobial use (55.8%) was the most reported element in primary care hospitals, followed by HAI point prevalence surveys (39.5%), and incidence surveillance of surgical site infection (SSI) (38.8%) (Table 1). No information was available on HAI incidence surveillance in neonatal intensive care units. The most frequently audited NHCPRC element in primary care hospitals was waste management (62.9%), followed by sterilization and medical device decontamination (58.3%), and environmental culturing (57.1%) (Table 1). Incidence surveillance of SSI (71.9%) was the most reported surveillance element in secondary/tertiary care hospitals, followed by point prevalence surveys (67.2%), and surveillance of antimicrobial resistance (64.3%) (Table 1). The most frequently audited NHCPRC element in secondary/tertiary care hospitals was environmental culturing (92.5%), followed by waste management (57.6%), and sterilization and medical device decontamination (55.3%) (Table 1).

### NHCPRC area “Education and training in IPC”

In addition to the above-mentioned survey reports, the search terms addressing the NHCPRC area on “Education and training in IPC” identified 5 single centre interventional studies: two non-controlled before-after studies, two controlled before-after studies, and one non-controlled interrupted time-series study. The quality of all five interventional studies was high (Additional file 1: Table S3C). Table 3 summarizes the details of the studies. Education and training in IPC was delivered via lectures, problem-based learning, (focus) group discussion, video scenarios, and simulation training. Two studies targeted new staff, whereas one focused on nursing students. Training activities were associated with improvement of IPC knowledge, increase of hand hygiene compliance, and reduction of HAIs.

### NHCPRC area “outcome and process indicator surveillance”

The search terms addressing the NHCPRC area on “outcome and process indicator surveillance” identified 17 observational studies (Table 2) and 7 interventional studies (Table 4). Of the 17 observational studies, 7 and 10 were of high and moderate quality, respectively (Table 2). All seven interventional studies were of high quality (Additional file 1: Table S3C). Five of the interventional studies applied a multimodal strategy, and measured either outcome indicators (*n* = 3) or process indicators (*n* = 2) (Table 4).

#### Observational studies on outcome- and process indicator surveillance

Table 2 summarizes the findings of the 17 observational studies: five measured device-associated HAIs, three all-cause HAIs, three *Clostridium difficile* infections (CDI), two ventilator-associated pneumonias (VAPs), two central line-associated bloodstream infections (CLABSIs), one SSIs, and one hospital acquired pneumonia. Twelve studies applied the standard Chinese surveillance protocol, four applied a network protocol other than the official document, and one applied a research protocol.

#### Interventional studies on outcome and process indicator surveillance

Table 4 summarizes the details of the 7 interventional studies: six non-controlled before-after studies and one randomized controlled trial. Due to variation in intervention and outcome measurement, no meta-analysis was performed. Five studies used a multimodal strategy addressing hand hygiene improvement, CLABSI prevention, and VAP prevention. One study successfully tested pre-operative chlorhexidine mouthwash on VAP reduction, whereas another study reported MRSA reduction in the environment by improved cleaning practices.

### Mapping to key/core components in IPC

This systematic review found information on all three NHCPRC areas, which are directly linked to the three ECDC key components on structure and organisation of IPC programmes, education and training in IPC, and performing surveillance (in a network) with timely feedback [6, 7]. However, many of the survey reports also reported on elements linked to other ECDC key components such as provision and appropriate promotion of (locally adapted) guidelines, and performing audits. Furthermore, some of the interventional studies improved the provision of alcohol-based handrub at the point of care, or used new catheter insertion kits and trolleys, which are elements linked to the “materials and ergonomics” ECDC key component. Most of the interventional studies applied a multimodal strategy. Together, this systematic review identified information on 7 of the 10 ECDC key components, and 7 of the 8 WHO core components, respectively (Table 5).

**Table 5:** Comparison with ECDC key components and WHO core components – Systematic review on implementation of infection prevention and control in acute care hospitals in Mainland China, 2012–2017

	NHCPRC areas [9]	Current systematic review	Core components (WHO) [7]	Key components (ECDC) [6]
Structure, organisation and management of IPC programmes	√	√	√	√
Provision and promotion of IPC guidelines	√	√	√	√
IPC education and training	√	√	√	√
Outcome and process indicator surveillance	√	√	√	√
Monitoring/auditing of IPC practices with individual feedback	√	√	√	√
Application of multimodal intervention strategies	N/A	√	√	√
Built environment, materials and equipment for IPC	N/A	√	√	√
Workload, staffing and bed occupancy	N/A	N/A	√	√
Engagement of champions	N/A	N/A	N/A	√
Positive organizational culture	N/A	N/A	N/A	√

Note: N/A: Not available information after data searching; *NHCPRC* National Health Commission of the People’s Republic of China

## Discussion

To our best knowledge, this is the first systematic review summarizing adoption and implementation of IPC in acute care hospitals in Mainland China. This review fills a research gap on the adoption and implementation of IPC in Mainland China (Table 6), highlighting, which of the key/core components recommended by ECDC/WHO have been adopted and implemented, and which need further attention. It also offers an overview on the distribution of strategies and elements available in primary care hospitals compared to secondary/tertiary care hospitals. The observational and interventional studies complete the findings of the survey reports, offering a more granular picture on IPC activities in acute care hospitals in Mainland China. To various degrees, there is evidence that seven of the ECDC key components have been adopted and implemented in acute care hospitals in Mainland China.

**Table 6:** Gaps of the three NHCPRC focus areas – Systematic review on implementation of infection prevention and control in acute care hospitals in Mainland China, 2012–2017

1. Structure, organisation and management of infection prevention and control - Limited IPC budget for IPC programmes; - High IPC staff turnover, particularly among IPC doctors; - Limited recognition by hospital management; - Limited feedback of the results to healthcare professionals.	
2. Education and training in infection prevention and control - Limited resources for IPC education and training; - Little experience with team-and task-oriented learning, or peer-to-peer teaching education; - Little experience with implementation strategies;	
3. Surveillance of outcome and process indicators - Few prospective incidence surveillance programmes - Little antimicrobial stewardship programmes in primary-care hospitals; - Little effort towards targeted MDRO screening of patients on admission	

*IPC* infection prevention and control, *NHCPRC* National Health Commission of the People’s Republic, *MDRO* multidrug-resistant microorganism

### Structure, organisation and management of infection prevention and control

Effective IPC in an acute care hospital needs an IPC programme with sufficient staffing and an allocated budget, support from the hospital management, and well defined duties and targets. The official structure requirements for IPC in Chinese hospitals (Additional file 1: Figure S1) are often not met. Only two-thirds of primary care hospitals have an IPC programme and an IPC committee. No information was found for any of the other elements of this NHCPRC area. The majority of secondary/tertiary care hospitals have an IPC programme, but only a third has an allocated budget. It is difficult to estimate the challenges on the proper functioning of IPC, but it has been shown that competing resources may have a negative impact on effective IPC [14]. Staffing, as identified in several reports is at minimal level [6, 7, 15], comparable to other surveys [16, 17]. However, high staff turnover, particularly among IPC doctors [18–20], is even of more concern than understaffing. This is partially explained by low salaries and limited career tracks [19]. Only 4.4% of IPC doctors were satisfied with their position in one survey [19]. They were assigned to that position by hospital management, often against their will [19]. As a result, most IPC departments are managed by junior IPC doctors and IPC nurses. Due to the hierarchical gap between doctors and nurses, as well as between junior and senior doctors, IPC professionals face structural challenges, and struggle in influencing behaviour change [21].

Almost all secondary/tertiary care hospitals have an IPC committee in place. However, the importance of IPC is not always recognized by hospital management, and IPC committee members do not always participate actively in the committees [19, 20]. Most secondary/tertiary care hospitals indicated to have a feedback mechanism in place. This is positive given the various IPC activities in surveillance and auditing. However, no details are available about format, target population, and frequency of feedbacks. Too often, information is conveyed to the hospital management only, and data reach healthcare workers too late, if at all, to be meaningful and impactful [22, 23].

A low proportion of primary care hospitals indicated to have IPC guidelines. This is surprising given that various national IPC documents are available (*n* = 30), and that implementation is mandatory for many of them (*n* = 17) (Additional file 1: Table S5).

### Education and training in infection prevention and control

Half of primary- and three-quarters of secondary/tertiary care hospitals indicated to have postgraduate IPC education and training in place. Education and training should be team- and task-oriented, frontline workers should take part in the preparation and execution process (ideally peer-to-peer teaching), the content should follow local guidelines, and implementation should be multimodal [6]. Unfortunately, survey reports lack details on the target population, contents, delivery methods, and frequency. Thus, it is difficult to assess the available resources for education and training and whether they are adequate. The use of multimodal strategies was identified in five interventional studies that successfully reduced HAIs by improving IPC knowledge and increasing hand hygiene compliance [24–28]. Professionals working in IPC need knowledge and skills on management and implementation research [29]. With the “European Certificate for Infection Control”, the European Society for Clinical Microbiology and Infectious Diseases has created a platform to offer comprehensive IPC training beyond hygiene to doctors [29, 30]. Implementation research and management are not part of IPC training in Mainland China. The basic and intermediate skill levels focus on legal aspects, mandatory surveillance, definitions, diagnosis, HAI classification, HAI prevention, and hand hygiene (Additional file 1: Tables S6A and S6B) [31]. The contents of the advanced level were not sufficiently specified in the documents to allow conclusions on delivering skills regarding on the topic of project management and implementation research [31].

### Surveillance of outcome and process indicators

#### Prevalence surveys, incidence surveillance of outcome- and process indicators

A range of surveillance activities were identified in the survey reports and in the observational studies, with significant differences between the hospital types. Many hospitals perform regular prevalence surveys, but given that yearly prevalence surveys on HAI are mandatory in Mainland China, the proportion of primary- (40%) and secondary/tertiary care hospitals (60%) is surprisingly low. Most prospective incidence surveillance measures SSI, which is similar to a recent European survey [22].

Survey reports do not specify device-association (such as CLABSI; CAUTI; or VAP); and there is no prospective CDI surveillance. This is particularly interesting considering that the 17 observational studies reported on VAP or other ventilator-associated events (*n* = 10) [32–41], CLABSI (*n* = 9) [32, 34–36, 38, 40–43], CAUTI (*n* = 6) [32, 34–36, 40, 41], and CDI (*n* = 3) [44–46]. The absence of detailing device-associated HAI-types and specifying CDI in regular surveillance reports is of concern taking into account the fact that problems, particularly with CDI (13–25 CDI/10,000 patient-days) [44, 46], were identified by some of the observational studies.

Not even two thirds of the hospitals perform surveillance on antimicrobial resistance and antimicrobial use, although this is mandatory since 2011 (Additional file 1: Table S5) [47, 48]. This is of concern given the challenge of emerging resistance in the Asia Pacific region [1, 3]. In addition, no information was identified on antimicrobial stewardship, which, at least according to some reports, is not yet established in primary care hospitals [3, 49]. In 2016, the CHINET surveillance programme reported 7.0% of Enterobacteriaceae being resistant to carbapenems, 38.4% of *Staphylococcus aureus* being resistant to methicillin, and 45.2% of Enterobacteriaceae expressing extended-spectrum beta-lactamases [50]. These numbers are alarming, and IPC should be empowered both in recognition and resources to prevent cross-transmission of multidrug-resistant microorganisms in acute care hospitals.

#### Monitoring/auditing

Standard and isolation precaution measures, waste management, sterilization and medical device decontamination, and environmental cultures are audited. Interestingly, there is no difference in the frequencies between the hospital types except for environmental cultures. Almost all secondary/tertiary care hospitals perform routine environmental cultures (in intensive care, operating theatres, central sterile services departments, endoscopy suites, haemodialysis centres, and dentistry departments), which is of questionable value outside of outbreaks [51]. These resources could be better directed towards targeted screening of patients at risk of carrying multidrug-resistant microorganisms [52].

#### Bundles and multimodal interventions

Consistent with the ECDC key components [6], five of the seven interventional studies used a multimodal strategy to improve hand hygiene, and reduce CLABSI and VAP [53–57]. Two studies on hand hygiene combined leadership engagement, provision of alcohol-based handrub at the point of care, feedback, and reminders at the workplace [53, 54]. One study on CLABSI prevention [55] and two studies on VAP prevention [56, 57] applied “bundles”, and partially followed the recommendations from SHEA/IDSA [58, 59]. These studies were performed in Eastern China, where the socioeconomic status is high, and therefore more resources might be available for implementing IPC measures and conducting studies (Additional file 1: Table S7) [3].

### Limitations

This review has limitations. First, the data on the first NHCPRC area about IPC structure, organisation and management came from survey reports using questionnaires. This limits detailing, and the proportion of hospitals correctly implementing IPC elements may be overestimated. Second, the survey reports and studies in the final analysis originated mainly from regions with higher socioeconomic status, and thus, may not be representative for Mainland China as a whole. Third, publication bias may have occurred by the fact that we checked only scientific data sources and confined search on one English and one Chinese database. Fourth, there is no mandatory reporting system for HAI incidence in Mainland China. Studies on incidence surveillance are mainly retrospective. Thus, data on outcome indicators are limited. Fifth, methodological heterogeneity of the observational and interventional studies limited comparability; and thus, conducting a formal meta-analysis was not possible. However, the aim of this systematic review on describing adoption and implementation of elements of the three NHCPRC areas was still met. Sixth, the search terms of this systematic review were based on the three NHCPRC areas. We considered the concept of the ECDC key components too new to serve as a starting point for a search based on scientific literature. The search strategy based on recommendations by the Chinese healthcare authorities was a more pragmatic approach. However, the results still covered 7 of the 10 ECDC key components, and 7 of the 8 WHO core components. The only lacking key components were about frontline staffing, integrating champions in the implementation of IPC strategies, and fostering a positive organisational culture.

## Conclusion

To variable degrees, there is evidence on implementation of all NHCPRC areas and of most of the ECDC key components and the WHO core components in acute care hospitals in Mainland China. The results are encouraging, but gaps in effective IPC were identified that may be used to guide future national policy-making in Mainland China.

## Additional file

Additional file 1: **Table S1A and S1B.** Search terms for PubMed and the China National Knowledge Infrastructure (Chinese database). **Table S2.** Definition of hospital types. **Table S3A and S3B.** Strengthening the Reporting of Observational Studies in Epidemiology checklist for survey reports and observational studies. **Table S3C and S3D.** Integrated Quality Criteria for Review of Multiple Study Designs for interventional studies. **Table S4A and S4B.** Survey reports of primary care hospitals and secondary−/tertiary care hospitals. **Table S5.** Chinese national guidelines related to infection prevention and control, 2009–2018. **Table S6A and S6B.** Details of education and training programme on infection prevention and control in Mainland China. **Table S7.** Geographical distribution of survey reports, observational studies and interventional studies in the final analysis. **Figure S1.** Organisation and structure on infection prevention and control in Chinese hospitals (Three levels). (DOCX 180 kb)

## Abbreviations

CAUTI: Catheter-associated urinary tract infection
CDI: *Clostridium difficile* infection
CLABSI: Central line-associated bloodstream infection
ECDC: European Centre for Disease Prevention and Control
HAI: Healthcare-associated infection
IPC: Infection prevention and control
MRSA: Methicillin-resistant *Staphylococcus aureus*
NHCPRC: National Health Commission of the People’s Republic of China
SIGHT: Systematic Review and Evidence-based Guidance on Organisation of Hospital Infection Control
SSI: Surgical site infection
VAP: Ventilator-associated pneumonia
WHO: World Health Organization
