# Supplementary material for: Implementation of infection prevention and control in acute care hospitals in Mainland China – a systematic review
Source: Antimicrob Resist Infect Control. 2019 Feb 11;8:32. doi: 10.1186/s13756-019-0481-y (PMC6371478; doi:10.1186/s13756-019-0481-y)
Supplement: Supplementary file 1 — Table S1A and S1B. Search terms for PubMed and the China National Knowledge Infrastructure (Chinese database). Table S2. Definition of hospital types. Table S3A and S3B. Strengthening the Reporting of Observational Studies in Epidemiology checklist for survey reports and observational studies. Table S3C and S3D. Integrated Quality Criteria for Review of Multiple Study Designs for interventional studies. Table S4A and S4B. Survey reports of primary care hospitals and secondary−/tertiary care hospitals. Table S5. Chinese national guidelines related to infection prevention and control, 2009–2018. Table S6A and S6B. Details of education and training programme on infection prevention and control in Mainland China. Table S7. Geographical distribution of survey reports, observational studies and interventional studies in the final analysis. Figure S1. Organisation and structure on infection prevention and control in Chinese hospitals (Three levels). (DOCX 180 kb) [file 13756_2019_481_MOESM1_ESM.docx]

**SUPPLEMENTARY MATERIAL**

**Implementation of infection prevention and control in acute care hospitals in Mainland China – A systematic review**

- **Supplementary table 1A and 1B** Search terms for PubMed and China National Knowledge Infrastructure (Chinese database)
- **Supplementary table 2** Definitions of hospital types
- **Supplementary table 3A and 3B** Strengthening the Reporting of Observational Studies in Epidemiology checklist for survey reports and observational studies
- **Supplementary table 3C and 3D** Integrated Quality Criteria for Review of Multiple Study Designs for interventional studies
- **Supplementary table 4A and 4B** Survey reports of primary care hospitals and secondary/tertiary care hospitals
- **Supplementary table 5** Chinese national guidelines related to infection prevention and control, 2009 – 2018
- **Supplementary table 6A and 6B** Details of education and training programme on infection prevention and control in Mainland China
- **Supplementary table 7** Geographical distribution of survey reports, observational studies and interventional studies in the final analysis
- **Supplementary figure 1** Organisation and structure on infection prevention and control in Chinese hospitals (Three levels)
- **References**

**Additional file 1: Table S1A Search terms for PubMed** - Implementation of infection prevention and control in acute care hospitals in Mainland China: a systematic review, 2012 – 2017

| **Concept 1A (Hospital organization, management, structure)**  Organization and management [MeSH terms] OR  Hospital management OR  Organizational development OR  Organizational structure OR  Personnel management [MeSH terms] OR  Program development [MeSH terms] OR  Hospital administration [MeSH terms] OR  Staff development [MeSH terms] OR  Risk management [MeSH terms] | **Concept 1B (Education and training)**  Education [MeSH terms] OR  Infection control education OR  Training | **Concept 1C (Surveillance)**  Hospital Surveillance OR  Population surveillance [MeSH terms] OR  Epidemiological Monitoring [MeSH terms] OR  Medical audit [MeSH terms] OR  Management audit [MeSH terms] OR  Clinical audit [MeSH terms] OR  Feedback [MeSH terms] |
| --- | --- | --- |
| **Concept 2 (infection control)**  Hospital infection OR  Cross infection [MeSH terms] OR  Infection control [MeSH terms] OR  Infection prevention OR  Prevention and control [MeSH terms] OR  Nosocomial infection OR  Healthcare-associated infection OR  Catheter-related infection [MeSH terms] OR  Catheterization, central venous [MeSH terms] OR  Catheter infection OR  Catheter-associated infection OR  Methicillin resistant staphylococcus aureus [MeSH terms] OR  Clostridium difficile [MeSH terms] OR  Vancomycin resistant enterococcus OR  Vancomycin resistant enterococci [MeSH terms] OR  Bacteremia [MeSH terms] OR  Pneumonia, ventilator-associated [MeSH terms] OR  Hand washing OR  Hand disinfection [MeSH terms] OR  Hand hygiene [MeSH terms] | | |
| **Concept 3 (Country)**  China [MeSH terms] | | |

Note: The search terms were through reference to the SIGHT study [[1](#_ENREF_1)].

**Additional file 1: Table S1B Search terms for China National Knowledge Infrastructure (Chinese database)** - Implementation of infection prevention and control in acute care hospitals in Mainland China: a systematic review, 2012 – 2017

| **Hospital management** | **Education and training** | **Surveillance** |
| --- | --- | --- |
| (Topic = healthcare-associated infection OR Topic = Nosocomial infection) AND (Topic = management OR Topic = Organization OR Topic = structure) | (Topic = healthcare-associated infection OR Topic = Nosocomial infection) AND (Topic = education OR Topic = training) | (Topic = healthcare-associated infection OR Topic = Nosocomial infection) AND (Topic = surveillance OR Topic = audit OR Topic = Feedback) |

**Additional file 1: Table S2** Definitions of hospital types - Implementation of infection prevention and control in acute care hospitals in Mainland China: a systematic review, 2012 – 2017

|  | **Primary care hospitals** | **Secondary care hospitals** | **Tertiary care hospitals** |
| --- | --- | --- | --- |
| Number of Hospital-beds | 20 – 100 | 101 – 500 | ≥ 500 |
| Ratio of hospital-beds to staffing | 1: 1-1.4 | 1: 1.3-1.5 | No report available |
| Ratio of doctor to nurse | No report available | 1:2 | 1:2 |
| Medical departments | Outpatient clinic, emergency, general internal medicine, general surgery, pediatrics, gynecology and obstetrics, ear-nose-throat (ENT) disease, and traditional Chinese medicine | Outpatients clinic, emergency, internal medicine (including respiratory diseases, gastrointestinal diseases, cardiology, endocrinology and nephrology), surgery (general surgery, orthopedics, urology), pediatrics, gynecology and obstetrics, ear-nose-throat (ENT) disease, intensive care (adult) | Outpatients clinic, emergency, internal medicine (including respiratory diseases, gastrointestinal diseases, cardiology, endocrinology, nephrology, hematology, oncology, and neurology), surgery (including general surgery, cardiac surgery, vascular surgery, neurosurgery, orthopedics, urology, plastic surgery, and burn units), gynecology and obstetrics, ear-nose-throat (ENT) diseases, pediatrics, neonatology, intensive care, and other departments (including traditional Chinese medicine, psychiatry, occupational health) |
| Services offered | The medical services include preventive medical health service (vaccination service), primary care service, outpatient-care service and rehabilitation service in one local community. Also patients with severe illness are referred to secondary-and tertiary-care hospital | The medical services include secondary-care service, outpatient-care service in more than one community. Also patients with severe illness are referred to tertiary-care hospital, specialty care hospital | The medical services include tertiary-care services, outpatients-care service in a city or even a province. Also it provides the medical training and scientific research. |

Note: The information was derived from standard for hospital classification management in China in 2006 [[2](#_ENREF_2)].

**Additional file 1: Table S3A** Strengthening the Reporting of Observational Studies in Epidemiology (STROBE) checklist [[3](#_ENREF_3)] for survey reports

| **Parameters** | **Item** | **Criteria** | | **Yes/No** |
| --- | --- | --- | --- | --- |
| **Introduction** |  |  | |  |
| Background/rationale | 1 | The study describes hospital organization, management and structure for infection prevention and control (IPC) in China | |  |
| Objectives | 2 | To provide an inventory on any report on adopting, implementing or analyzing one or more IPC indicators at acute-care facility level in Chinese hospitals | |  |
| **Methods** |  |  | |  |
| Study design | 3 | Structure questionnaire survey that is summarized the number of hospitals in the context of applying one or more IPC indicators | |  |
| Settings | 4 | Primary care hospitals (PCHs), and secondary/tertiary care hospitals (STCHs) | |  |
| Participants | 5 | Hospitals | |  |
| Variables | 6 | Author, publication year, province in China, numbers of potential indicators concerning about IPC activities (e.g. structure, organization and management of IPC, IPC education and training, and surveillance of outcome and process indicators) | |  |
| Data sources | 7 | Investigators actively collect the data sources, which is reported by each individual hospital | |  |
| Bias | 8 | The study provides information on assessment of bias | |  |
| Study sample size | 9 | Hospitals participating the surveys are stratified by PCHs and STCHs | |  |
| Statistical methods | 10 | The study explains applied statistical methods:   - Describes the proportion of potential indicators - Statistical difference of indicators between PCHs and STCHs | |  |
| **Results** |  |  | |  |
| Descriptive data | 11 | The study was reported the proportions of indicators (e.g. structure, organization and management of IPC, IPC education and training, and surveillance of outcome and process indicators) | |  |
| **Discussion** |  |  | |  |
| Key results | 12 | Key results are summarized with reference to study objectives | |  |
| Limitations | 13 | Limitations are sufficiently discussed | |  |
| Interpretation | 14 | Overall interpretation of results is based on the findings and in the context of the evidence base | |  |
| Generalizability | 15 | Generalisability (external validity) of the study results is discussed | |  |
| **Total** |  |  | |  |
| **Rating** | | | **Description** | |
| High quality | | | Fulfilled >75% of STROBE criteria | |
| Moderate quality | | | Fulfilled 50-75% of STROBE criteria | |
| Low quality | | | Fulfilled <50% of STROBE criteria | |

**Additional file 1: Table S3B** Strengthening the Reporting of Observational Studies in Epidemiology (STROBE) checklist [[3](#_ENREF_3)] for observational studies

| **Parameters** | **Item** | **Criteria** | | **Yes/No** |
| --- | --- | --- | --- | --- |
| **Introduction** |  |  | |  |
| Background/rationale | 1 | The study describes the importance of prospective incidence surveillance of healthcare-associated infections (HAIs) in acute-care facility in China | |  |
| Objectives | 2 | To determine the incidence rate of HAIs in acute-care facility | |  |
| **Methods** |  |  | |  |
| Study design | 3 | Prospective incidence surveillance | |  |
| Settings | 4 | Acute-care facility | |  |
| Participants | 5 | Hospitalized adults, children and/or neonates | |  |
| Variables | 6 | Author, publication year, province in China, study aim, setting, surveillance protocol, sample size, study duration, methodology, and outcome | |  |
| Data sources | 7 | Investigators actively collect data from the patient’s medical file or hospital (electronic) information system | |  |
| Bias | 8 | The study provides information on assessment of bias | |  |
| Study sample size | 9 | The study reports sample sizes for the acute-care facility | |  |
| Statistical methods | 10 | The study explains applied statistical methods to describe the analysis of overall incidence rate | |  |
| **Results** |  |  | |  |
| Descriptive data | 11 | The study reports the incidence rate of HAIs | |  |
| **Discussion** |  |  | |  |
| Key results | 12 | Key results are summarized with reference to study objectives | |  |
| Limitations | 13 | Limitations are sufficiently discussed | |  |
| Interpretation | 14 | Overall interpretation of results is based on the findings and in the context of the evidence base | |  |
| Generalizability | 15 | Generalizability (external validity) of the study results is discussed | |  |
| **Total** |  |  | |  |
| **Rating** | | | **Description** | |
| High quality | | | Fulfilled >75% of STROBE criteria | |
| Moderate quality | | | Fulfilled 50-75% of STROBE criteria | |
| Low quality | | | Fulfilled <50% of STROBE criteria | |

**Additional file 1: Table S3C** Integrated Quality Criteria for Review of Multiple Study Designs (ICROMS) for interventional studies [[4](#_ENREF_4)]

| Dimension | Specific criteria | Chen S [[5](#_ENREF_5)] | He M [[6](#_ENREF_6)] | Li Q [[7](#_ENREF_7)] | Zhang Y [[8](#_ENREF_8)] | Mu X [[9](#_ENREF_9)] | Su D [[10](#_ENREF_10)] | Zhou Q [[11](#_ENREF_11)] | Lin Y [[12](#_ENREF_12)] | Huang M [[13](#_ENREF_13)] | Zhao L [[14](#_ENREF_14)] | Zhou Q [[15](#_ENREF_15)] | Tao L [[16](#_ENREF_16)] |
| --- | --- | --- | --- | --- | --- | --- | --- | --- | --- | --- | --- | --- | --- |
| Study design |  | NCITS | NCBA | NCBA | CBA | NCBA | NCBA | NCBA | RCT | CBA | NCBA | NCBA | NCBA |
| 1. Clear aims and justification | A. Clear statement of the aims of the research? | ✓ | ✓ | ✓ | ✓ | ✓ | ✓ | ✓ | ✓ | ✓ | ✓ | ✓ | ✓ |
|  | B. Rationale for number of pre- and post-intervention points or adequate baseline measurement | ✓ | ✓ | ✓ | ✗ | ✓ | ✓ | ✓ | ? | ✗ | ✓ | ✓ | ✓ |
|  | C. Explanation for lack of control group | ✗ | ✗ | ✗ | ✗ | ✗ | ✗ | ✗ | ✗ | ✗ | ✗ | ✗ | ✗ |
|  | D. Appropriateness of qualitative methodology | ✗ | ✗ | ✗ | ✗ | ✗ | ✗ | ✗ | ✗ | ✗ | ✗ | ✗ | ✗ |
|  | E. Appropriate study design | ? | ✗ | ? | ✗ | ? | ? | ? | ? | ✗ | ✗ | ? | ? |
| 2. Managing bias in sampling or between group | A. Sequence generation | ✗ | ✗ | ✗ | ✗ | ✗ | ✗ | ✗ | ✓ | ✗ | ✗ | ✗ | ✗ |
|  | B. Allocation concealment | ✗ | ✗ | ✗ | ✗ | ✗ | ✗ | ✗ | ✓ | ✗ | ✗ | ✗ | ✗ |
|  | C. Justification for sample choice | ✓ | ✓ | ✓ | ✗ | ✓ | ✓ | ✓ | ✗ | ✗ | ✓ | ✓ | ✓ |
|  | D. Intervention and control group selection designed to protect against systematic difference or Selection bias | ✗ | ✗ | ✗ | ✓ | ✗ | ✗ | ✗ | ✗ | ✓ | ✗ | ✗ | ✗ |
|  | E. Comparability of groups | ✗ | ✗ | ✗ | ✗ | ✗ | ✗ | ✗ | ✗ | ✗ | ✗ | ✗ | ✗ |
|  | F. Sampling and recruitment | ✗ | ✗ | ✗ | ✗ | ✗ | ✗ | ✗ | ✗ | ✗ | ✗ | ✗ | ✗ |
| 3. Managing bias in outcome measurements and blinding | A. Blinding | ✗ | ✗ | ✗ | ✗ | ✗ | ✗ | ✗ | ✓ | ✗ | ✗ | ✗ | ✗ |
|  | B. Baseline measurement – protection against selection bias | ✗ | ✗ | ✗ | ✓ | ✗ | ✗ | ✗ | ✗ | ✓ | ✗ | ✗ | ✗ |
|  | C. Protection against contamination | ✗ | ✗ | ✗ | ✓ | ✗ | ✗ | ✗ | ✗ | ✓ | ✗ | ✗ | ✗ |
|  | D. Protection against secular changes | ✗ | ✗ | ✗ | ✗ | ✗ | ✗ | ✗ | ✗ | ✗ | ✗ | ✗ | ✗ |
|  | E. Protection against detection bias: blinded assessment of primary outcome measures | ✓ | ✓ | ✓ | ✓ | ✓ | ✓ | ✓ | ✓ | ✓ | ✓ | ✓ | ✓ |
|  | F. Reliable primary outcome measures | ✓ | ✓ | ✓ | ✓ | ✓ | ✓ | ✓ | ✓ | ✓ | ✓ | ✓ | ✓ |
|  | G. Comparability of outcomes | ✗ | ✗ | ✗ | ✗ | ✗ | ✗ | ✗ | ✗ | ✗ | ✗ | ✗ | ✗ |
| 4. Managing bias in follow-up | A. Follow-up of subjects (protection against exclusion bias) | ✗ | ✗ | ✗ | ✗ | ✗ | ✗ | ✗ | ✓ | ✗ | ✗ | ✗ | ✗ |
|  | B. Follow-up of patients or episodes of care | ✗ | ✗ | ✗ | ✗ | ✗ | ✗ | ✗ | ✓ | ✗ | ✗ | ✗ | ✗ |
|  | C. Incomplete outcome data addressed | ✓ | ✓ | ✓ | ✓ | ✓ | ✓ | ✓ | ✓ | ✓ | ✓ | ✓ | ✓ |
| 5. Managing bias in other study aspects | A. Protection against detection bias: intervention unlikely to affect data collection | ✓ | ✓ | ✓ | ✓ | ✓ | ✓ | ✓ | ✓ | ✓ | ✓ | ✓ | ✓ |
|  | B. Protection against information bias | ✗ | ✗ | ✗ | ✗ | ✗ | ✗ | ✗ | ✗ | ✗ | ✗ | ✗ | ✗ |
|  | C. Data collection appropriate to address research aims | ✗ | ✗ | ✗ | ✗ | ✗ | ✗ | ✗ | ✗ | ✗ | ✗ | ✗ | ✗ |
|  | D. Attempts to mitigate effects of no control | ✓ | ✓ | ✓ | ✗ | ✓ | ✓ | ✓ | ✗ | ✗ | ✓ | ✓ | ✓ |
| 6. Analytical rigor | A. Sufficient data points to enable reliable statistical inference | ✗ | ✗ | ✗ | ✗ | ✗ | ✗ | ✗ | ✗ | ✗ | ✗ | ✗ | ✗ |
|  | B. Shaping of intervention effect specified | ✗ | ✗ | ✗ | ✗ | ✗ | ✗ | ✗ | ✗ | ✗ | ✗ | ✗ | ✗ |
|  | C. Analysis sufficiently rigorous/free from bias | ✗ | ✓ | ✓ | ✓ | ✓ | ✓ | ✓ | ✓ | ✓ | ✓ | ✓ | ✓ |
| 7. Managing bias in reporting/ethical consideration | A. Free of selective outcome reporting | ✓ | ✓ | ✓ | ✓ | ✓ | ✓ | ✓ | ✓ | ✓ | ✓ | ✓ | ✓ |
|  | B. Limitations addressed | ✗ | ✗ | ✓ | ✗ | ✓ | ✓ | ✓ | ✓ | ✗ | ✗ | ✓ | ✓ |
|  | C. Conclusions clear and justified | ✓ | ✓ | ✓ | ✓ | ✓ | ✓ | ✓ | ✓ | ✓ | ✓ | ✓ | ✓ |
|  | D. Free of other bias | ✓ | ✓ | ✓ | ✓ | ✓ | ✓ | ✓ | ✓ | ✓ | ✓ | ✓ | ✓ |
|  | E. Ethics issues addressed | ✗ | ✗ | ✓ | ✗ | ✓ | ✓ | ✓ | ✓ | ✗ | ✗ | ✓ | ✓ |
| Final ICROMS score |  | 23 | 24 | 29 | 24 | 29 | 29 | 28 | 34 | 24 | 24 | 29 | 29 |
| Quality |  | High | High | High | High | High | High | High | High | High | High | High | High |

**Additional file 1: Table S3D** Decision matrix – mandatory criteria and minimum score for study type to be included in review

| Study design | Mandatory criteria | Minimum score |
| --- | --- | --- |
| RCT, cRCT | 1A, 2A, 2B and 3A | 22 |
| CBA | 1A, 2D, 3B and 3C | 18 |
| CITS | 1A, 3D and 6A | 18 |
| NCITS | 1A, 1B, 2C and 5D | 22 |
| NCBA | 1A, 1B, 2C and 5D | 22 |
| Cohort | 1A, 2E, 3G and 4C | 18 |
| Qualitative | 1A, 1E and 2F | 16 |

Note: Studies must meet mandatory criteria and a minimum score.

Study designs: CBA = controlled before-after; CITS = controlled interrupted time series; cRCT = cluster-randomized control trial; NCITS = non-controlled interrupted time series; NCBA = non-controlled before-after; RCT = randomized controlled trial;

Note: ICROMS: Integrated Quality Criteria for Review of Multiple Study Designs; Scores applicable to each criteria: “Yes” (Criterion met) gained 2 points; “Unclear” (unclear whether or not the criterion is met) gained 1 point; “No” (criterion not met) gained 0 point.

Note: Low quality: 0 – 12 points; medium quality: 13 – 20 points; and high quality: ≥21 points.

**Additional file 1: Table S4A** Survey reports of primary care hospitals

| **No.** | **Author** | **Year** | **Province** | **Quality** | Tot | Gui | Organisation and structure | | | | | Edu | Surveillance | | | | | | Auditing | | | |
| --- | --- | --- | --- | --- | --- | --- | --- | --- | --- | --- | --- | --- | --- | --- | --- | --- | --- | --- | --- | --- | --- | --- |
|  |  |  |  |  | A | B | C | D | E | F | G | H | I | J | K | L | M | N | O | P | Q | R |
| 1 | Chen H, et al [[17](#_ENREF_17)] | 2017 | Jilin | Moderate | 152 | 104 | 146 | 122 |  |  |  | 87 | 92 | 73 | 50 |  | 60 | 105 |  | 129 | 122 | 115 |
| 2 | Xia Q, et al [[18](#_ENREF_18)] | 2017 | Chongqing | Low | 43 |  | 28 | 1 |  |  |  |  |  |  |  |  |  |  |  |  |  |  |
| 3 | Hua C, et al [[19](#_ENREF_19)] | 2016 | Henan | Moderate | 36 | 13 |  |  |  |  |  | 25 |  |  |  |  |  | 24 | 31 | 33 | 31 | 11 |
| 4 | Zhang Q, et al [[20](#_ENREF_20)] | 2016 | Shanghai | Moderate | 44 | 43 | 44 | 44 |  |  |  | 41 |  |  |  |  | 23 |  |  | 42 |  | 43 |
| 5 | Xing J, et al [[21](#_ENREF_21)] | 2015 | Xinjiang | Moderate | 40 | 5 | 3 |  |  |  |  | 18 |  |  |  |  |  |  | 10 | 0 | 13 |  |
| 6 | Xu T, et al [[22](#_ENREF_22)] | 2015 | Anhui | Moderate | 36 | 9 | 25 |  |  |  |  | 18 | 0 | 0 | 0 |  | 0 | 0 | 0 | 14 | 9 | 0 |
| 7* | Wang H, et al [[23](#_ENREF_23)] | 2014 | Henan | Moderate | 7 |  |  |  |  |  |  | 3 |  |  |  |  |  | 0 |  | 3 |  |  |
| 8 | Chen Y, et al [[24](#_ENREF_24)] | 2014 | Hunan | Moderate | 44 | 10 |  | 2 |  |  |  |  |  |  |  |  |  |  | 40 | 39 | 40 | 35 |
| 9* | Hao M, et al [[25](#_ENREF_25)] | 2013 | Sichuan | Moderate | 19 |  |  | 18 |  |  |  | 3 |  |  |  |  |  |  |  | 6 | 2 |  |
| 10 | Du F, et al [[26](#_ENREF_26)] | 2012 | Hebei | Low | 45 | 45 | 10 |  |  |  |  | 8 | 0 |  |  |  | 0 |  | 0 | 0 | 0 | 0 |

Note: Tot: Total number of participating hospitals; Gui: IPC guidelines; Edu: IPC education and training.

Note: A. Number of hospitals; B. Provision of adapted IPC guidelines; C. IPC committee; D. Formal IPC programme; E: Feedback of IPC indicators; F: Allocated IPC funding/budgets; G: Activities in IPC research; H: Regular IPC training; I: Point prevalence surveys; J: Incidence surveillance of surgical site infections; K: Incidence surveillance in intensive care units; L: Incidence surveillance in neonate intensive care units; M: Surveillance antimicrobial resistance; N: Surveillance of antimicrobial consumption; O: Standard precautions and isolation precautions; P: Healthcare waste management; Q: Sterilization and medical device decontamination; R: Environmental culturing.

Note: * Wang H, et al [[23](#_ENREF_23)] and Hao M, et al [[25](#_ENREF_25)] studies contribute to the database both primary care hospitals (PCHs) and secondary/tertiary care hospitals (STCHs). The blank of the cell represents no information. Number of “0” in the cell represents that the hospital did not practice this IPC activity.**Additional file 1: Table S4B** Survey reports of secondary/tertiary care hospitals

| **No.** | **Author** | **Year** | **Province** | **Quality** | Tot | Gui | Organisation and structure | | | | | Edu | Surveillance | | | | | | Auditing | | | | IPC staffing | | | | | |
| --- | --- | --- | --- | --- | --- | --- | --- | --- | --- | --- | --- | --- | --- | --- | --- | --- | --- | --- | --- | --- | --- | --- | --- | --- | --- | --- | --- | --- |
|  |  |  |  |  | A | B | C | D | E | F | G | H | I | J | K | L | M | N | O | P | Q | R | S | T | U | V | W | X |
| 1 | Hu Q, et al [[27](#_ENREF_27)] | 2017 | Hubei | Low | 47 |  |  |  |  |  |  |  |  |  |  |  | 47 |  |  |  |  |  | 57996 | 192 | 45 | 96 | 151 |  |
| 2 | Sun J, et al [[28](#_ENREF_28)] | 2017 | Sichuan | Moderate | 18 | 15 |  |  |  |  |  | 10 | 10 | 10 | 10 | 10 | 10 | 10 | 7 | 12 | 10 | 18 |  |  |  |  |  |  |
| 3 | Zhang Y, et al [[29](#_ENREF_29)] | 2017 | Hunan | Moderate | 85 |  | 83 | 77 |  |  |  |  |  |  |  |  |  |  |  |  |  |  | 31376 | 128 | 10 | 117 | 51 | 20 |
| 4 | Zhang Z, et al [[30](#_ENREF_30)] | 2017 | Heilongjiang | Moderate | 60 | 57 | 60 | 50 |  |  | 9 |  |  |  |  |  |  |  |  |  |  |  | 33278 | 187 | 42 | 129 | 111 | 77 |
| 5 | Zhang Z, et al [[31](#_ENREF_31)] | 2017 | Shaanxi | Low | 29 |  |  |  |  |  |  |  |  |  |  |  |  |  |  |  |  |  |  | 46 | 7 | 30 | 22 | 11 |
| 6 | Wang L, et al [[32](#_ENREF_32)] | 2016 | Hubei | Moderate | 84 | 83 | 82 | 48 | 84 | 14 |  |  |  |  |  |  |  |  |  |  |  |  | 18970 |  |  |  |  |  |
| 7 | Liu F, et al [[33](#_ENREF_33)] | 2016 | Shaanxi | High | 165 | 162 | 157 | 158 | 145 | 56 | 71 |  | 119 | 123 | 57 | 39 | 114 |  |  |  |  | 151 | 73387 | 394 |  |  |  |  |
| 8 | Liu W, et al [[34](#_ENREF_34)] | 2016 | Inner Mongolia | Moderate | 143 |  | 143 | 127 |  |  | 26 |  |  | 117 | 57 | 44 | 89 | 90 |  |  |  |  |  |  |  |  |  |  |
| 9 | Huang S, et al [[35](#_ENREF_35)] | 2016 | Fujian | Moderate | 47 |  |  |  |  |  |  |  |  | 21 | 15 | 7 | 19 |  |  |  |  |  |  | 123 | 11 | 87 | 59 | 54 |
| 10 | Shen L, et al [[36](#_ENREF_36)] | 2016 | Hubei | Moderate | 63 | 62 | 59 |  | 63 |  | 5 |  |  |  |  |  |  |  |  |  |  |  | 14257 | 141 | 42 | 86 | 36 | 32 |
| 11 | Ding L, et al [[37](#_ENREF_37)] | 2016 | Xinjiang | Moderate | 101 |  | 101 | 101 |  |  |  | 99 |  |  |  |  |  |  |  |  |  |  |  | 217 | 37 | 143 | 87 |  |
| 12* | Wang H, et al [[23](#_ENREF_23)] | 2014 | Henan | Moderate | 21 |  |  | 14 |  |  |  | 17 |  |  |  |  | 10 | 14 | 5 | 8 |  |  |  |  |  |  |  |  |
| 13 | Mu X, et al [[38](#_ENREF_38)] | 2014 | Guizhou | Moderate | 102 | 43 | 101 | 65 |  |  |  | 46 |  |  |  |  |  |  |  |  |  |  | 31535 | 212 | 65 | 129 | 71 | 60 |
| 14 | Zhang J, et al [[39](#_ENREF_39)] | 2014 | Anhui | Moderate | 18 |  | 18 | 14 |  |  |  | 14 | 6 |  |  |  | 6 |  |  |  |  | 17 |  |  |  |  |  |  |
| 15 | Zhong Y, et al [[40](#_ENREF_40)] | 2014 | Sichuan | Low | 23 |  |  |  |  |  |  |  |  |  |  |  |  |  |  |  |  |  | 26801 | 105 | 43 | 42 | 59 | 47 |
| 16 | Li J, et al [[41](#_ENREF_41)] | 2013 | Shaanxi | Moderate | 33 |  |  | 32 |  | 16 | 15 | 25 |  | 21 | 18 |  |  |  |  |  |  |  |  | 81 | 19 | 53 | 47 |  |
| 17 | Zhang Y, et al [[42](#_ENREF_42)] | 2013 | Gansu | Low | 48 |  |  |  |  |  |  |  |  |  |  |  |  |  |  |  |  |  |  | 133 | 46 | 62 | 53 | 59 |
| 18 | Li Y, et al [[43](#_ENREF_43)] | 2013 | Guangxi | Moderate | 61 |  | 61 | 61 |  |  |  | 61 |  |  |  |  |  |  |  |  |  |  |  |  |  |  |  |  |
| 19* | Hao M, et al [[25](#_ENREF_25)] | 2013 | Sichuan | Moderate | 20 |  |  | 14 |  |  |  | 11 |  |  |  |  |  |  |  | 14 | 11 |  |  |  |  |  |  |  |

Note: Tot: Total number of participating hospitals; Gui: IPC guidelines; Edu: IPC education and training.

Note: A. Number of hospitals; B. Provision of adapted IPC guidelines; C. IPC committee; D. Formal IPC programme; E: Feedback of IPC indicators; F: Allocated IPC funding/budgets; G: Activities in IPC research; H: Regular IPC training; I: Point prevalence surveys; J: Incidence surveillance of surgical site infections; K: Incidence surveillance in intensive care units; L: Incidence surveillance in neonate intensive care units; M: Surveillance antimicrobial resistance; N: Surveillance of antimicrobial consumption; O: Standard precautions and isolation precautions; P: Healthcare waste management; Q: Sterilization and medical device decontamination; R: Environmental culturing; S: Hospital-beds; T: IPC professionals; U: IPC doctors; V: IPC nurses; W: IPC professionals with high education level; X: Senior IPC professionals.

Note: * Wang H, et al [[23](#_ENREF_23)] and Hao M, et al [[25](#_ENREF_25)] studies contribute to the database both primary care hospitals (PCHs) and secondary/tertiary care hospitals (STCHs). The blank of the cell represented no information available.

**Additional file 1 : Table S5** Chinese national guidelines related to infection prevention and control, 2009 – 2018

| **No.** | **Title** | **Date of publication** | **Date of implementation** | **Mandatory** | **voluntary** |
| --- | --- | --- | --- | --- | --- |
| 1 | Regulation for prevention and control of healthcare associated infections in outpatient department and emergency department in healthcare facilities (WS/T 591 - 2018) [[44](#_ENREF_44)] | 2018-05-10 | 2018-11-01 |  | √ |
| 2 | Accreditation regulation of control and prevention of healthcare-associated infections in hospitals (WS/T 592 - 2018) [[45](#_ENREF_45)] | 2018-05-10 | 2018-11-01 |  | √ |
| 3 | Procedure for blood culture collection and processing (WS/T 503 – 2017) [[46](#_ENREF_46)] | 2017-09-06 | 2018-03-01 |  | √ |
| 4 | The basic function specification of healthcare-associated infection management information system (WS/T 547 - 2017) [[47](#_ENREF_47)] | 2017-07-25 | 2017-12-01 |  | √ |
| 5 | Regulation for prevention and control of healthcare-associated infections of airborne transmission disease in healthcare facilities (WS/T 511 – 2016) [[48](#_ENREF_48)] | 2016-12-27 | 2017-06-01 |  | √ |
| 6 | Guidelines for infection prevention and control in general ward in healthcare facilities (WS/T 510 – 2016) [[49](#_ENREF_49)] | 2016-12-27 | 2017-06-01 |  | √ |
| 7 | Regulation for prevention and control of healthcare-associated infections in intensive care unit (WS/T 509 – 2016) [[50](#_ENREF_50)] | 2016-12-27 | 2017-06-01 |  | √ |
| 8 | Regulation for cleaning and disinfection of environmental surfaces in healthcare facilities (WS/T 512 – 2016) [[51](#_ENREF_51)] | 2016-12-27 | 2017-06-01 |  | √ |
| 9 | Regulation for disinfection and sterilization of dental instruments (WS 506 – 2016) [[52](#_ENREF_52)] | 2016-12-27 | 2017-06-01 | √ |  |
| 10 | Regulation for cleaning and disinfection of flexible endoscopes (WS 507 – 2016) [[53](#_ENREF_53)] | 2016-12-27 | 2017-06-01 | √ |  |
| 11 | Central sterile supply department (CSSD) – Part 1: Management standard WS 310.1 – 2016 (Updated) [[54](#_ENREF_54)] | 2016-12-27 | 2017-06-01 | √ |  |
| 12 | Central sterile supply department (CSSD) – Part 2: Standard operating procedures for cleaning, disinfection and sterilization WS 310.2 – 2016 (Updated) [[55](#_ENREF_55)] | 2016-12-27 | 2017-06-01 | √ |  |
| 13 | Central sterile supply department (CSSD) – Part 3: Surveillance standard for cleaning, disinfection and sterilization WS 310.3 – 2016 (Updated) [[56](#_ENREF_56)] | 2016-12-27 | 2017-06-01 | √ |  |
| 14 | Guideline for professional training and management of healthcare-associated  Infections (WS/T 525 – 2016) [[57](#_ENREF_57)] | 2016-08-02 | 2017-01-15 |  | √ |
| 15 | Guideline of control of healthcare-associated infection outbreak (WS/T 524 – 2016) [[58](#_ENREF_58)] | 2016-08-02 | 2017-01-15 |  | √ |
| 16 | Basic requirements for healthcare-associated infections management in primary care facilities [[59](#_ENREF_59)] | 2013-12-23 | 2013-12-23 | √ |  |
| 17 | Detailed rules for the implementation of accreditation standards for tertiary care hospitals (2011 version) [[60](#_ENREF_60)] | 2013-12-23 | 2013-12-23 | √ |  |
| 18 | Action plan for prevention and control of healthcare associated infections (2012 – 2015) [[61](#_ENREF_61)] | 2012-09-25 | 2012-09-25 | √ |  |
| 19 | Hygienic standard for disinfection in hospitals (GB 15982 – 2012) [[62](#_ENREF_62)] | 2012-06-29 | 2012-11-01 | √ |  |
| 20 | The management approach for the administration of antibiotics [[63](#_ENREF_63)] | 2012-04-24 | 2012-08-01 | √ |  |
| 21 | Air handling management in healthcare facilities (WS/T 368 – 2012) [[64](#_ENREF_64)] | 2012-04-05 | 2012-08-01 |  | √ |
| 22 | Guideline for prevention and control of healthcare-associated infections due to multidrug-resistant organisms [[65](#_ENREF_65)] | 2011-01-17 | 2011-01-17 | √ |  |
| 23 | Guideline for prevention and control of catheter-related urinary tract infections [[66](#_ENREF_66)] | 2010-11-29 | 2010-11-29 | √ |  |
| 24 | Guideline for prevention and control of central line-associated bloodstream infections [[67](#_ENREF_67)] | 2010-11-29 | 2010-11-29 | √ |  |
| 25 | Guideline for prevention and control of surgical site infections [[68](#_ENREF_68)] | 2010-11-29 | 2010-11-29 | √ |  |
| 26 | Guideline for the diagnosis and treatment of NDM-1 multidrug resistant Enterobacteriaceae Bacteria [[69](#_ENREF_69)] | 2010-09-28 | 2010-09-28 | √ |  |
| 27 | Standard for healthcare-associated infections surveillance (WS/T 312 – 2009) [[70](#_ENREF_70)] | 2009-04-01 | 2009-12-01 | √ |  |
| 28 | Standard for hand hygiene among healthcare workers in healthcare facilities (WS/T 313 – 2009) [[71](#_ENREF_71)] | 2009-04-01 | 2009-12-01 |  | √ |
| 29 | Healthcare-associated infection outbreak, management and reports [[72](#_ENREF_72)] | 2009-07-20 | 2009-07-20 | √ |  |
| 30 | Guideline for prevention and control of occupational exposure to bloodborne pathogen (GBZ/T 213 – 2008) [[73](#_ENREF_73)] | 2009-03-02 | 2009-09-01 |  | √ |

**Additional file 1: Table S6A** Basic level education and training programme on infection prevention and control in Mainland China [[57](#_ENREF_57)]

| **Topic** | **Outline** | **Teaching approach** |
| --- | --- | --- |
| Legal regulations | 1. Law of the People's Republic of China on prevention and control of infectious diseases;  2. Medical waste management;  3. Medical institution administration and management;  4. Management of healthcare-associated infections;  5. Disinfection and sterilization. | 1.On-site professional training;  2.Self-learning;  3. E-learning. |
| Theories | 1. Organizational structure of the IPC department;  2. Hospital high-risk departments for healthcare-associated infections (e.g. ICU, NICU);  3. Definition and types of healthcare-associated infections;  4. Common pathogens encountered in healthcare-associated infections;  5. Diagnosis and prevention of healthcare-associated infections;  6. Cleaning, disinfection and sterilization;  7. Hand hygiene. | 1.On-site professional training;  2.Self-learning;  3. E-learning. |
| Basic skills | 1. Surveillance of healthcare-associated infections – purposes and significance;  2. Surveillance of healthcare-associated infections – definition, calculation, data collection;  3. Surveillance of healthcare-associated infections – management;  4. Guidelines of healthcare-associated infections surveillance – contents, interventions, and effectiveness;  5. Hospital environmental hygiene surveillance (air, devices, surfaces);  6. Hand Hygiene  7. Common disinfection and sterilization methods, and the monitoring of the effectiveness;  8. Standard and isolation precaution measures. | 1.On-site professional training;  2.Self-learning;  3. E-learning. |
| Exercises | Demonstrate proficiency at performing three commonly encountered procedures | Direct observation |

Note: **Basic training** (first phase) is recommended for **new staff and IPC staff** with less than 2 years of experience. It covers the laws and regulations, basic theory, and basic skills.

**Additional file 1 : Table S6B** Intermediate level education and training programme on infection prevention and control in Mainland China [[57](#_ENREF_57)]

| **Topic** | **Outline** | **Teaching approach** |
| --- | --- | --- |
| Legal regulations | New and revised law and regulations. | 1. E-learning;  2. Self-learning. |
| Theory | 1. Antimicrobial stewardship;  2. Monitoring of healthcare-associated infections in high-risk departments:   - Intensive care; - Neonatology; - Gynecology and obstetrics; - Operating theatres; - Central sterilization; - Endoscopy suite; - Haemodialysis center; - Dentistry. | 1.On-site professional training;  2. E-learning |
| Advanced skills | 1. Prevention of healthcare-associated infections   - Surgical site infections - Central line-associated bloodstream infections - Catheter-associated urinary tract infections - Ventilator-associated pneumonia   2. Infections in transplant patients  3. Infections due to multidrug-resistant microorganisms  4. Communicable diseases | 1.On-site professional training  2. E-learning |
| Exercises | Demonstrate proficiency at performing surveillance on three types of healthcare-associated infections | Direct observation, simulation training, and group discussion |

Note: **Intermediate training** (second phase) is recommended for **IPC staff** with 2-5 years of experience. It focuses on hospital surveillance, and outbreak recognition, investigation and control.

Note: **Advanced training** (third phase) is recommended for **IPC staff** with at least 5 years of experience. It involves the acquisition of new knowledge and skills for staff education and conducting IPC related research. No details were given in the guideline for this phase.

**Additional file 1 : Table S7** Geographical distribution of survey reports, observational studies and interventional studies in the final analysis

| Region | Province/ Municipality | Organisation and structure data | | Interventional studies(N) | Surveillance data (N) | HAI prevalence  %(95%CI) | GDP per capita (CNY) | Population density(million) |
| --- | --- | --- | --- | --- | --- | --- | --- | --- |
|  |  | STCH(N) | PCH(N) |  |  |  |  |  |
| Northern region | Beijing |  |  |  | 1 | 1.73 (0.80-2.66) | 106,497 | 21.7 |
|  | Tianjin |  |  |  |  | 3.37 (3.17-3.59) | 107,960 | 15.6 |
|  | Hebei |  |  | 1 |  | 3.89 (2.34-5.43) | 40,255 | 74.7 |
|  | Shanxi |  |  |  |  | 4.93 (1.69-8.17) | 34,919 | 36.8 |
|  | Inner Mongolia | 1 |  |  | 1 | 2.06 (1.82-2.31) | 71,101 | 25.2 |
| Northeast region | Liaoning |  |  |  | 1 |  | 65,354 | 43.8 |
|  | Jilin |  | 1 |  |  |  | 51,086 | 27.3 |
|  | Heilongjiang | 1 |  |  |  |  | 39,462 | 38.0 |
| Eastern region | Shanghai |  | 1 | 3 | 3 | 3.73 (3.44-4.03) | 103,796 | 24.2 |
|  | Jiangsu |  |  |  | 3 | 2.26 (1.57-2.95) | 87,995 | 79.9 |
|  | Zhejiang |  |  | 1 | 1 | 3.73 (1.74-5.72) | 77,644 | 55.9 |
|  | Anhui | 1 | 1 |  | 1 | 1.99 (1.91-2.07) | 35,997 | 62.0 |
|  | Fujian | 1 |  | 2 |  | 3.47 (3.32-3.63) | 67,966 | 38.7 |
|  | Jiangxi |  |  |  |  | 3.85 (1.52-6.18) | 36,724 | 45.9 |
|  | Shandong |  |  |  |  | 2.05 (1.45-2.64) | 64,168 | 99.5 |
| Central region | Henan | 1 | 2 |  |  | 4.23 (3.21-5.24) | 39,123 | 95.3 |
|  | Hubei | 3 | 1 |  | 1 | 3.51 (3.18-3.84) | 50,654 | 58.9 |
|  | Hunan | 1 | 1 |  |  | 4.92 (3.88-5.96) | 42,754 | 68.2 |
| Southern region | Guangdong |  |  | 1 |  | 2.28 (1.66-2.90) | 67,503 | 109.9 |
|  | Guangxi | 1 |  |  |  | 2.34 (1.42-3.25) | 35,190 | 48.4 |
|  | Hainan |  |  |  |  | 5.45 (4.68-6.30) | 40,818 | 9.2 |
| Southwest region | Chongqing |  | 1 |  |  | 4.82 (4.53-5.12) | 52,321 | 30.5 |
|  | Sichuan | 3 | 1 |  | 2 | 2.72 (2.37-3.08) | 36,775 | 82.6 |
|  | Guizhou | 1 |  | 2 |  | 2.78 (2.38-3.19) | 29,847 | 35.6 |
|  | Yunnan |  |  | 1 |  | 3.64 (1.20-6.08) | 28,806 | 47.7 |
|  | Tibet |  |  |  |  |  | 31,999 | 3.3 |
| Northwest region | Shaanxi | 3 |  |  |  |  | 47,626 | 38.1 |
|  | Gansu | 1 |  |  |  |  | 26,165 | 26.1 |
|  | Qinghai |  |  |  |  |  | 41,252 | 5.9 |
|  | Ningxia |  |  |  |  |  | 43,805 | 6.8 |
|  | Xinjiang | 1 | 1 |  |  | 2.87 (1.69-4.05) | 40,036 | 23.9 |
|  | Multi-region |  |  | 1 | 3 |  |  |  |
|  | Total | 19 | 10 | 12 | 17 | 3.12 (2.94-3.29) |  | 1382.7 |

Note: PCH: Primary-care hospital; STCH: Secondary/tertiary hospital.

No data (Organisation and structure data, interventional studies, surveillance data, and prevalence data) was available from Tibet Autonomous Region, Qinghai Province and Ningxia Hui Autonomous Region. HAI prevalence data were derived from Wang and colleagues’ publication of “The prevalence of healthcare-associated infections in mainland China: a systematic review and meta-analysis”[[74](#_ENREF_74)]; The GDP per capita and population densities in China were derived from China Statistical Yearbook 2016, which was compiled by National Bureau of Statistics of China [[75](#_ENREF_75)]. The empty cells represent no available publication or relevant data; Two studies in IPC organisation and structure contributed to the database both primary care hospitals (PCHs) and secondary/tertiary care hospitals (STCHs).

**Additional file 1: Figure S1** Organisation and structure on infection prevention and control in Chinese hospitals (Three levels)

Note: CSSD: Central sterile services department; IPC: Infection prevention and control.

IPC organization and structure is applicable for acute healthcare facilities with ≥100 hospital-beds; This figure is adapted from the guideline of “*Nosocomial Infection Management Method”* (Decree No. 48) published by the ministry of health of the People’s Republic of China in 2006, along with Chinese national guidelines related to infection prevention and control.

**REFERENCES**

1. Zingg W, Holmes A, Dettenkofer M, Goetting T, Secci F, Clack L, et al. Hospital organisation, management, and structure for prevention of health-care-associated infection: a systematic review and expert consensus. Lancet Infect Dis. 2015;15:212-224.

2. National Health Commission of the People’s Republic of China. Standard for hospital classification management in China (in Chinese). 2006.

3. von Elm E, Altman DG, Egger M, Pocock SJ, Gotzsche PC, Vandenbroucke JP. The Strengthening the Reporting of Observational Studies in Epidemiology (STROBE) statement: guidelines for reporting observational studies. Lancet. 2007;370:1453-1457.

4. Zingg W, Castro-Sanchez E, Secci FV, Edwards R, Drumright LN, Sevdalis N, et al. Innovative tools for quality assessment: integrated quality criteria for review of multiple study designs (ICROMS). Public Health. 2016;133:19-37.

5. Chen S, Han G, Li L, Xiong X. Training at morning shift meeting can improve awareness rate of healthcare-associated infection knowledge among health care workers (in Chinese). Chin J Infect Control. 2017;16:858-861.

6. He M, Lin X, Zeng H, Fang W, Lin X. Effect of pre-job training about healthcare-associated infections at military hospitals (in Chinese). J Prev Med Chin PLA. 2017;35:28-30.

7. Li QF, Xu H, Ni XP, Lin R, Jin H, Wei LY, et al. Impact of relocation and environmental cleaning on reducing the incidence of healthcare-associated infection in NICU. World J Pediatr. 2017;13:217-221.

8. Zhang Y, Zheng D, Tian B, Chen X, Zhang S, Xu J. The effectiveness of training in improving the infection prevention and control knowledge among new healthcare professionals (in Chinese). Shenzhen J Integrated Traditional Chin and Western Med. 2016;26:177-178.

9. Mu X, Xu Y, Yang T, Zhang J, Wang C, Liu W, et al. Improving hand hygiene compliance among healthcare workers: an intervention study in a Hospital in Guizhou Province, China. Braz J Infect Dis. 2016;20:413-418.

10. Su D, Hu B, Rosenthal VD, Li R, Hao C, Pan W, et al. Impact of the International Nosocomial Infection Control Consortium (INICC) Multidimensional Hand Hygiene Approach in five intensive care units in three cities of China. Public Health. 2015;129:979-988.

11. Zhou Q, Lee SK, Hu XJ, Jiang SY, Chen C, Wang CQ, et al. Successful reduction in central line-associated bloodstream infections in a Chinese neonatal intensive care unit. Am J Infect Control. 2015;43:275-279.

12. Lin YJ, Xu L, Huang XZ, Jiang F, Li SL, Lin F, et al. Reduced occurrence of ventilator-associated pneumonia after cardiac surgery using preoperative 0.2% chlorhexidine oral rinse: results from a single-centre single-blinded randomized trial. J Hosp Infect. 2015;91:362-366.

13. Huang M, Xu J, Zhao Y, Chen Y. Effect of IPC training conducted by infection prevention and control professionals on hand hygiene compliance among nursing interns (in Chinese). J Bethune Med Sci. 2014;12:608-609.

14. Zhao L, Yang R, Yuan H. Role of hospital infection knowledge training in improving aseptic techniques of medical workers (in Chinese). Chin J Nosocomiol. 2014;24:3082-3084.

15. Zhou Q, Lee SK, Jiang SY, Chen C, Kamaluddeen M, Hu XJ, et al. Efficacy of an infection control program in reducing ventilator-associated pneumonia in a Chinese neonatal intensive care unit. Am J Infect Control. 2013;41:1059-1064.

16. Tao L, Hu B, Rosenthal VD, Zhang Y, Gao X, He L. Impact of a multidimensional approach on ventilator-associated pneumonia rates in a hospital of Shanghai: findings of the International Nosocomial Infection Control Consortium. J Crit Care. 2012;27:440-446.

17. Chen H, Li J, Luo N, Kong X, Li H, Yang S, et al. Investigation on current situation of nosocomial infection management in county level medical institutions in Jilin province (in Chinese). Med and Society. 2017;30:30-34.

18. Xia Q, Gu Z, Shen S, Tan X, Wu Y, Li S. Investigation on current situation of nosocomial infection management in primary-care institutions in Chongqing City (in Chinese). J Chongqing Med. 2017;46:1410-1411.

19. Hua C, Han Z, Li Y, Liu X, Liu C, Wen J. Current situation of healthcare-associated infection management in 36 primary medical institutions in Henan province (in Chinese). Chin J Infect Control. 2016;15:757-759.

20. Zhang Q, Zheng J, Zou C, Xu J, Jiang J. Current status of control and management of nosocomial infections in 44 community health service centers (in Chinese). Chin J Nosocomiol. 2016;26:4283-4285.

21. Xing J, Shang H. Investigation on current situation of nosocomial infection management in 40 primary-care institutions (in Chinese). J Xinjiang Med. 2015;45:777-778.

22. Xu T, Chen J, Song J, Wang R, Lv D. Investigation of infection management in primary hospital and improvement measures (in Chinese). Chin J General Practice. 2015;13:2004-2006.

23. Wang H, Jin L. Investigation and analysis of current situation of hospital infection management in 28 traditional Chinese medicine institutions (in Chinese). Clin J Traditional Chin Med. 2014;26:1353-1354.

24. Chen Y, Wen X, Fu C, Wu A, Ren N. Status of healthcare-associated infection management in the primary care medical institutes (in Chinese). Chin J Infect Control. 2014;13:556-559.

25. Hao M, Huang Y, Li L. Current status of control of nosocomial infections in 39 hospitals (in Chinese). Chin J Nosocomiol. 2013;23:3210-3212.

26. Du F, Wang Z. Investigation of infection control management in primary hospital and improvement measures (in Chinese). Med Research and Education. 2012;29:101-104.

27. Hu Q, Ming X, Xu Y, Liang J, Liu X, Xu M, et al. Present situation of healthcare-associated infection management and prevention and control of multidrug-resistant organisms in Hubei province (in Chinese). Chin J Infect Control. 2017;16:717-720.

28. Sun J, Wen L, He D. Current situation and countermeasure of nosocomial infection management in secondary and above medical institutions (in Chinese). J West Chin Med. 2017;32:1075-1079.

29. Zhang Y, Zhu X, Xiao Y. Status of healthcare-associated infection management professionals in 85 traditional Chinese medicine institutions (in Chinese). Chin J Infect Control. 2017;16:199-202.

30. Zhang Z, Cao Z, Shu D, Zhang L, Ma Y, Liu L, et al. Investigation of status of healthcare-associated infection management in 60 secondary-care hospitals in Harbin city (in Chinese). Chin J Disinfect. 2017;34:190-191.

31. Zhang Z, Han M, Yang Z, Wei Q, Wang X, Wei Q, et al. Current situation of full-time healthcare-associated infection management staff in Xianyang City of Shaanxi Province (in Chinese). Chin J Infect Control. 2017;16:635-638.

32. Wang L. Current situation and problems of infection management in secondary hospitals in Hubei province (in Chinese). Chin Health Standard Management. 2016;7:188-190.

33. Liu F, Dong H, Fan S, Wang H, Suo Y. Current situation of healthcare-associated infection management systems in Shaanxi Province (in Chinese). Chin J Infect Control. 2016;15:702-705.

34. Liu W, Hai Y, Jiao Y, Zhang K, Xu B, Bai H, et al. Current situation of healthcare-associated infection management in Inner Mongolia Autonomous Region (in Chinese). Chin J Infection Control. 2016;15:706-709.

35. Huang S. Investigation and analysis of current situation of hospital infection management of tertiary-and secondary-care hospitals in Quanzhou city (in Chinese). Chin Med and Pharmacy. 2016;6:179-181.

36. Shen L. Investigation and analysis of current situation of hospital infection management in 63 secondary-care hospitals in Hubei Province (in Chinese). Shenzhen J Integrated Traditional Chin and Western Med. 2016;26:183-184.

37. Ding L, Yu L, Wang P, Liu Y, Gulimire A. Development situation of healthcare-associated infection management organizations in Xinjiang region (in Chinese). Chin J Infection Control. 2016;15:710-713.

38. Mu X, Xu Y, Liu W, Wang C, Zhang J, Chen J, et al. Investigation on current situation of professional setting of nosocomial infection management in Guizhou province (in Chinese). Chin J Nosocomiol. 2014;24:5156-5157.

39. Zhang J, Zhou Q. Investigation on current situation of healthcare-associated infection management in 18 healthcare institutions (in Chinese). J Anhui Med. 2014;35:234-235.

40. Zhong Y, Xu S, Wang Y, Zong Z. Management of hospital infection control of 23 hospitals in Sichuan: a cross-sectional survey (in Chinese). Chin J Evid-based Med. 2014;14:174-177.

41. Li J, Liu Y, Qin X, Liu B, Zhuang Y, Chang F, et al. Investigation on nosocomial infection control in 33 hospitals in Shaanxi province (in Chinese). J Nurs Sci. 2013;28:86-88.

42. Zhang Y, Zhang H, Jin F, Zhang J. Current status of full-time staff of nosocomial infections control in Gansu province (in Chinese). Chin J Nosocomiol. 2013;23:3448-3449.

43. Li Y, Pang J, Lv L, Sang B, Zhao Y, Zhou X, et al. Current status of nosocomial infection control in traditional Chinese medicine hospitals (in Chinese). Chin J Nosocomiol. 2013;23:5010-5012.

44. National Health Commission of the People’s Republic of China. Regulation for prevention and control of healthcare associated infection in outpatient department and emergency department in healthcare facilities (WS/T 591 - 2018) (in Chinese). 2018. Available at: <http://www.moh.gov.cn/ewebeditor/uploadfile/2018/05/20180523150938396.pdf>. Accessed 22 October 2018.

45. National Health Commission of the People’s Republic of China. Accreditation regulation of control and prevention of healthcare-associated infection in hospital (WS/T 592 - 2018) (in Chinese). 2018. Available at: <http://www.nhfpc.gov.cn/ewebeditor/uploadfile/2018/05/20180523110555724.pdf>. Accessed 22 October 2018.

46. National Health Commission of the People’s Republic of China. Procedure for blood culture collection and processing (WS/T 503 – 2017) (In Chinese). 2017. Available at: <http://www.moh.gov.cn/ewebeditor/uploadfile/2017/10/20171024163700956.pdf>. Accessed 22 October 2018.

47. National Health Commission of the People’s Republic of China. The basic function specification of healthcare-associated infection management information system (WS/T 547 - 2017) (In Chinese). 2017. <http://wjw.nmg.gov.cn/uploadfiles/201806/27/2018062716285688558160.pdf>. Accessed 22 October 2018.

48. National Health Commission of the People’s Republic of China. Regulation for prevention and control of healthcare-associated infection of airborne transmission disease in healthcare facilities (WS/T 511 – 2016) (In Chinese). 2016. Available at: <http://www.moh.gov.cn/ewebeditor/uploadfile/2017/01/20170119150530360.pdf>. Accessed 22 October 2018.

49. National Health Commission of the People’s Republic of China. Guidelines for infection prevention and control in general ward in healthcare facilities (WS/T 510 – 2016) (In Chinese). 2016. Available at: <http://www.nhfpc.gov.cn/ewebeditor/uploadfile/2017/01/20170119150408703.pdf>. Accessed 22 October 2018.

50. National Health Commission of the People’s Republic of China. Regulation for prevention and control of healthcare-associated infection in intensive care unit (WS/T 509 – 2016) (In Chinese). 2016. Available at: <http://www.moh.gov.cn/ewebeditor/uploadfile/2017/01/20170119150236548.pdf>. Accessed 22 October 2018.

51. National Health Commission of the People’s Republic of China. Regulation for cleaning and disinfection of environmental surfaces in healthcare facilities (WS/T 512 – 2016) (In Chinese). 2016. Available at: <http://www.moh.gov.cn/ewebeditor/uploadfile/2017/01/20170119150706183.pdf>. Accessed 22 October 2018.

52. National Health Commission of the People’s Republic of China. Regulation for disinfection and sterilization of dental instruments (WS 506 – 2016) (In Chinese). 2016. Available at: <http://www.moh.gov.cn/ewebeditor/uploadfile/2017/01/20170119145649720.pdf>. Accessed 22 October 2018.

53. National Health Commission of the People’s Republic of China. Regulation for cleaning and disinfection of flexible endoscopes (WS 507 – 2016) (In Chinese). 2016. Available at: <http://www.moh.gov.cn/ewebeditor/uploadfile/2017/01/20170119145924475.pdf>. Accessed 22 October 2018.

54. National Health Commission of the People’s Republic of China. Central sterile supply department (CSSD) – Part 1: Management standard (WS 310.1 – 2016) (In Chinese). 2016. Available at: <http://www.nhfpc.gov.cn/ewebeditor/uploadfile/2017/01/20170105090443523.pdf>. Accessed 22 October 2018.

55. National Health Commission of the People’s Republic of China. Central sterile supply department (CSSD) – Part 2: Standard operating procedures for cleaning, disinfection and sterilization (WS 310.2 – 2016) (In Chinese). 2016. Available at: <http://www.nhfpc.gov.cn/ewebeditor/uploadfile/2017/01/20170105090606684.pdf>. Accessed 22 October 2018.

56. National Health Commission of the People’s Republic of China. Central sterile supply department (CSSD) – Part 3: Surveillance standard for cleaning, disinfection and sterilization (WS 310.3 – 2016) (In Chinese). 2016. Available at: <http://www.nhfpc.gov.cn/ewebeditor/uploadfile/2017/01/20170105090648964.pdf>. Accessed 22 October 2018.

57. Wu A, Huang X, Li L, Gong Y, Liu C, Wang L, et al. Guideline for professional training about managing of healthcare associated infections WS/T 525-2016 (in Chinese). Chin J Infect Control. 2017;16:94-97.

58. National Health Commission of the People’s Republic of China. Guideline of control of healthcare-associated infection outbreak (WS/T 524 – 2016) (In Chinese). 2016. Available at: <http://www.moh.gov.cn/ewebeditor/uploadfile/2016/08/20160815113050215.pdf>. Accessed 22 October 2018.

59. National Health Commission of the People’s Republic of China. Basic requirements for healthcare-associated infection management in primary-care facilities [National Health Administration Medical Development (2013) No. 40] (In Chinese). 2013. Available at: <http://www.nhfpc.gov.cn/yzygj/s3585/201312/0283f92d9c424a86b2ca6f625503b044.shtml>. Accessed 22 October 2018.

60. National Health Commission of the People’s Republic of China. Detailed rules for the implementation of accreditation standards for tertiary-care hospitals (2011 version) (In Chinese). 2011. Available at: <http://www.nhfpc.gov.cn/yzygj/s3585u/201112/06f754a213d8413787904e9e6439d88b.shtml>. Accessed 22 October 2018.

61. National Health Commission of the People’s Republic of China. Action plan for prevention and control of healthcare associated infection (2012 – 2015) (In Chinese). 2012. Available at: <http://www.moh.gov.cn/mohyzs/s3593/201210/56062.shtml>. Accessed 22 October 2018.

62. National Health Commission of the People’s Republic of China. Hygienic standard for disinfection in hospitals (GB 15982 – 2012) (In Chinese). 2012. Available at: <http://www.nhfpc.gov.cn/ewebeditor/uploadfile/2014/10/20141029163321351.pdf>. Accessed 22 October 2018.

63. National Health Commission of the People’s Republic of China. The management approach for the administration of antibiotics (Ministry of Health No. 84) (In Chinese) 2012. Available at: <http://www.gov.cn/flfg/2012-05/08/content_2132174.htm>. Accessed 22 October 2018.

64. National Health Commission of the People’s Republic of China. Air handling management in healthcare facilities (WS/T 368 – 2012) (In Chinese). 2012. Available at: <http://www.nhfpc.gov.cn/zwgkzt/s9496/201204/54511/files/8df30d0236d3421c87492786c55c26e7.pdf>. Accessed 22 October 2018.

65. National Health Commission of the People’s Republic of China. Guideline for prevention and control of healthcare-associated infections due to multidrug-resistant organisms (In Chinese). 2011. Available at: <http://www.moh.gov.cn/mohyzs/s3594/201101/50487.shtml>. Accessed 22 October 2018.

66. National Health Commission of the People’s Republic of China. Guideline for prevention and control of catheter-related urinary tract infection (Health Medical Administration No.187) (in Chinese). 2010. Available at: <http://www.moh.gov.cn/mohyzs/s3594/201012/50039.shtml>. Accessed 22 October 2018.

67. National Health Commission of the People’s Republic of China. Guideline for prevention and control of catheter-related bloodstream infection (Health Medical Administration No.187) (in Chinese). 2010. Available at: <http://www.moh.gov.cn/mohyzs/s3594/201012/50039.shtml>. Accessed 22 October 2018.

68. National Health Commission of the People’s Republic of China. Guideline for prevention and control of surgical site infection (Health Medical Administration No.187) (in Chinese). 2010. Available at: <http://www.moh.gov.cn/mohyzs/s3594/201012/50039.shtml>. Accessed 22 October 2018.

69. National Health Commission of the People’s Republic of China. Guideline for the diagnosis and treatment of NDM-1 multidrug resistant Enterobacteriaceae Bacteria (in Chinese). 2010. Available at: <http://www.moh.gov.cn/mohyzs/s8233/201010/49274.shtml>. Accessed 22 October 2018.

70. National Health Commission of the People’s Republic of China. Standard for nosocomial infection surveillance (in Chinese). 2009. Available at: <http://www.nhfpc.gov.cn/cmsresources/mohyzs/cmsrsdocument/doc5842.pdf>. Accessed 22 October 2018.

71. National Health Commission of the People’s Republic of China. Standard for hand hygiene among healthcare workers in healthcare facilities (WS/T 313 – 2009) (in Chinese). 2009. Available at: <http://www.moh.gov.cn/zwgkzt/s9496/200904/40118/files/5fe4afce5b874512a9780c724a4d5be0.pdf>. Accessed 22 October 2018.

72. National Health Commission of the People’s Republic of China. Healthcare-associated infection outbreak, management and reports (Health Medical Administration in 2009 No.73) (in Chinese). 2009. Available at: <http://www.moh.gov.cn/mohyzs/s3593/200907/41962.shtml>. Accessed 22 October 2018.

73. National Health Commission of the People’s Republic of China. Guideline for prevention and control of occupational exposure to bloodborne pathogen (GBZ/T 213 – 2008) (in Chinese). 2009. Available at: <http://www.moh.gov.cn/zwgkzt/pyl/200909/42930/files/f3beee0e56424ad1b7f5d09380155e73.pdf>. Accessed 22 October 2018.

74. Wang J, Liu F, Tartari E, Huang J, Harbarth S, Pittet D, et al. The Prevalence of Healthcare-Associated Infections in Mainland China: A Systematic Review and Meta-analysis. Infect Control Hosp Epidemiol. 2018;39:701-709.

75. Data of GDP per capita and indices in China: China Statistical Yearbook (in Chinese). 2016. China Staitstics Press website: <http://www.stats.gov.cn/tjsj/ndsj/2016/indexeh.htm>. Accessed 22 October 2018.
